# Effect of Auxins on the Accumulation of Alkaloids in Ungrafted *Annona emarginata* (Schltdl.) H. Rainer and *Annona emarginata* (Schltdl.) H. Rainer Grafted with *Annona atemoya* Mabb.

**DOI:** 10.3390/molecules30092070

**Published:** 2025-05-07

**Authors:** Carolina Ovile Mimi, Iván De-la-Cruz-Chacón, Felipe Moura Araujo da Silva, Victor Cauan Rocha Roberto, Gisela Ferreira

**Affiliations:** 1 Department of Biodiversity and Biostatistics, Institute of Biosciences, São Paulo State University (UNESP), Prof. Dr. Antônio Celso Wagner Zanin Street, 250, Botucatu 18618-689, SP, Brazil; carolina.mimi@unesp.br (C.O.M.); victor.rocha-roberto@unesp.br (V.C.R.R.); gisela.ferreira@unesp.br (G.F.); 2 Laboratorio de Fisiología y Química Vegetal, Instituto de Ciencias Biológicas, Universidad de Ciencias y Artes de Chiapas, Libramiento Norte-Poniente 1150, Tuxtla Gutiérrez 29039, CS, Mexico; 3 Multidisciplinary Support Center, Federal University of Amazonas (UFAM), General Rodrigo Octávio Avenue, 6200, Manaus 69080-900, AM, Brazil; felipemourams@gmail.com

**Keywords:** Annonaceae, liriodenine, indoleacetic acid, indolebutyric acid, naphthaleneacetic acid

## Abstract

Plant regulators, such as auxins, modulate the synthesis of specialized metabolites and aid in the bioprospection of molecules. *Annona emarginata* is known to produce antifungal alkaloids and serves as a rootstock for *Annona atemoya*. This study evaluated the effects of indoleacetic acid (IAA), indolebutyric acid (IBA), and naphthaleneacetic acid (NAA) applications on the accumulation of alkaloids in ungrafted *A. emarginata* and grafted with *A. atemoya*. Total alkaloids were analyzed by spectrophotometry, and alkaloid profiles were analyzed by DI-MS at 8, 14, and 20 days after treatments (DAT). The results indicated that IAA and NAA had the strongest effects on increasing the synthesis of alkaloids in the roots of ungrafted seedlings. In grafted plants, IBA had a more pronounced effect on roots; however, at final evaluation, all three auxins had an impact on both roots and leaves. Chemometric analysis revealed that auxins also altered the alkaloid composition in both seedling types. Nineteen alkaloids were identified regardless of treatment and harvest time. Eight alkaloids were identified for the first time in *A. emarginata* and nine were identified in *A. atemoya*. The main alkaloids found in ungrafted seedlings treated with IAA, IBA, and NAA were liriodenine and lanuginosine. In grafted seedlings, liriodenine and reticuline were the primary alkaloids found in roots, whereas liriodenine, lanuginosine, and reticuline were significantly present in leaves. The use of auxins to enhance alkaloid biosynthesis demonstrates their potential for bioprospection and the development of crops tolerant to biotic stress.

## 1. Introduction

The genus *Annona* (Annonaceae) is widely distributed in Brazil, with approximately 80 of the 160 species, 24 of which are endemic [1,2,3]. *Annona* stands out both for the production of fresh fruits [4,5] and for the synthesis of specialized metabolites such as alkaloids [6], which are considered the main specialized metabolites acting as chemotaxonomic markers of the genus. Alkaloids such as anonaine, asimilobine, liriodenine and reticuline are commonly found in *Annona* [7,8,9]. Alkaloids play an ecological role by influencing chemical interactions between plants and their environment [10,11,12,13]. They are also of significant interest in the search for bioactive molecules for use in the pharmaceutical and agrochemical industries [14,15,16]. For example, liriodenine and anonaine exhibit potential antibacterial, antifungal, antioxidant, anticancer, and antidepressant activities [17,18].

The biosynthesis of specialized metabolites, such as alkaloids, occurs through genetic control and can be modulated by different factors [19]. Among these factors, the supply of auxins has a varying impact on the modulation of alkaloids [20,21], which depends on the specific species and type of auxin. This variation can increase alkaloid synthesis, affecting both the quantity and concentration of molecules within the alkaloid profile [22,23,24]. Thus, evaluating the action of different auxins in the synthesis of alkaloids allows a greater understanding of the relationship between these molecules and their biosynthetic pathways, being a possible alternative for increasing the production of bioactive molecules in native and commercial plants, adding ecological and economic value to these species and helping to improve commercial production.

Among commercialized *Annonas*, *Annona atemoya* Mabb. (*Annona cherimola* Mill. × *Annona squamosa* L.) has been gaining prominence in food markets [5,25] due to its organoleptic properties such as sweet flavor (~31 °BRIX), pleasant aroma and texture, and high nutritional value (high K, Cu, Mg, and vitamin C content) [15,26,27]. One of the challenges for the production of atemoya (*A. atemoya*) is its susceptibility to fungal diseases, such as anthracnose (genus *Colletotrichum*) and root rot (*Rhizoctonia solani*, *Pythium* sp., and *Cylindrocladium clavatum*) [28]. One of the alternatives to mitigate such vulnerability is the use of production techniques such as grafting [29]. The choice of the appropriate rootstock is based on the evaluation of its compatibility with the scion and tolerance to abiotic and biotic stresses [29], and species with a high synthesis of specialized metabolites can provide greater tolerance of grafted plants to pathogens [30].

*Annona emarginata* (Schltdl.) H. Rainer, specifically ‘terra-fria’ morphotype, is native to Brazil and is one of the most commonly used rootstocks for *A. atemoya*. This species, known as ‘araticum de terra-fria’, is preferred for rootstock due to its anatomical and physiological compatibility [30,31], drought tolerance [32], resistance to borer attacks, and resistance to root rot [30]. In addition, *A. emarginata* produces a variety of alkaloids, primarily benzylisoquinolines, including liriodenine and anonaine [7,9,33].

In particular, it presents antifungal alkaloids against *Colletotrichum fructicola* and *Colletotrichum theobromicola*, which are fungi that cause anthracnose [33]. However, to date, there is no report of any study analyzing the effects of plant regulators on the synthesis of alkaloids in atemoya plants grafted onto *A. emarginata*.

In this context, the aim of this study was to evaluate whether different auxins (indoleacetic acid—IAA, indolebutyric acid—IBA and naphthaleneacetic acid—NAA) influence the synthesis of alkaloids in *Annona emarginata*, both ungrafted and grafted with *Annona atemoya*. These findings may have potential applications in the pharmaceutical industry or in the development of biotic stress-tolerant crops.

## 2. Results

### 2.1. Concentration of Total Alkaloids

Ungrafted *A. emarginata* seedlings, as those grafted with *A. atemoya*, produce alkaloids in roots and leaves, and in both types of seedlings, the root is the site of greatest production.

Auxins caused different changes in the concentration of total alkaloids compared to control (Table 1 and Table 2). In *A. emarginata* roots (ungrafted plant), IAA and NAA increase alkaloid production in the three evaluation periods (47–100% and 53–181%, respectively), while IBA induced a 66% increase only at 14 days after treatments (DAT) (Table 1). In *A. emarginata* roots grafted with *A. atemoya*, it was observed that IBA increased the amount of alkaloids by approximately 28 to 45% in the three evaluation times, while IAA and NAA stimulated alkaloid production (67% and 61%, respectively) only at 20 DAT.

In addition to the modulation caused by the supply of auxins, modulation is also observed as a result of grafting, where *A. emarginata* roots grafted with *A. atemoya* naturally present a greater production of alkaloids (20 to 70%) when compared with ungrafted *A. emarginata* roots. Similar behavior was observed with the supply of IBA, where greater production of total alkaloids is observed in the roots of seedlings used as rootstock (increase ranging from 34 to 87%) than in ungrafted *A. emarginata* roots (Table 1).

In *A. emarginata* leaves (ungrafted), only IAA led to an increase of approximately 38% in the production of total alkaloids at 20 DAT, while IBA reduced the concentration of total alkaloids by approximately 35% at 14 DAT and 24% at 20 DAT (Table 2). In contrast, in *A. atemoya* leaves (seedlings grafted onto *A. emarginata* roots), it was observed that IAA increased the concentration of alkaloids in the three evaluation times both in comparison to control (51–141% increase) and in comparison to IBA and NAA, which in turn increased the production of total alkaloids in the three evaluation times (increases of 17–51% and 8–63%, respectively) in relation to the control.

### 2.2. Alkaloid Profile by DI-MS and Chemometric Analysis

**Figure 1:**
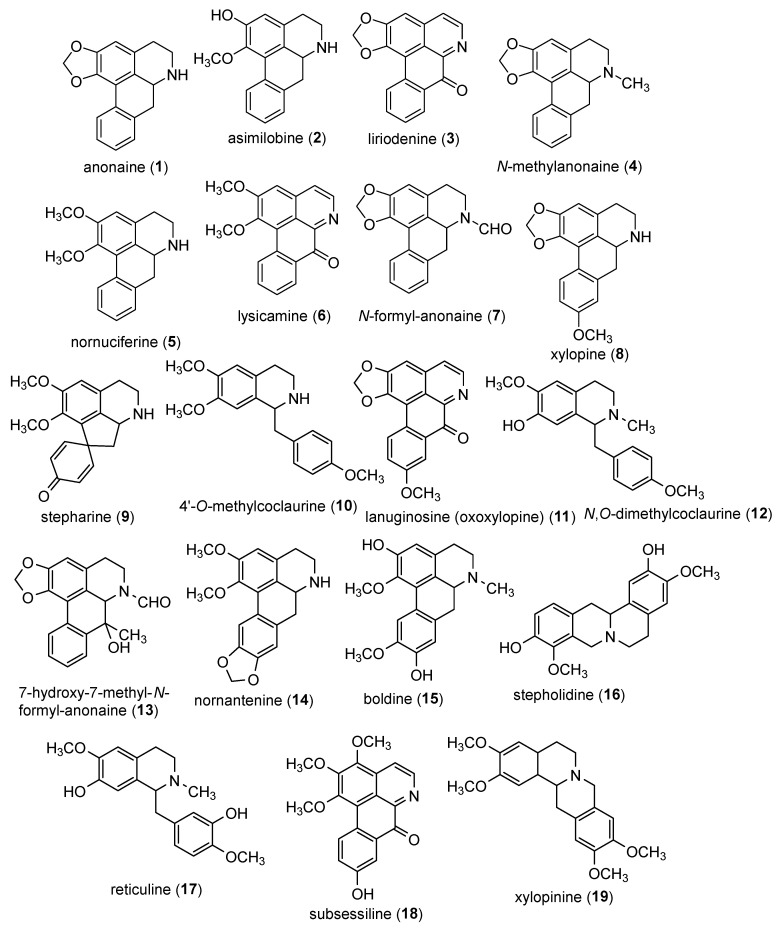
Alkaloids identified in roots and leaves of ungrafted *Annona emarginata* and *A. emarginata* grafted with *Annona atemoya* treated with IAA, IBA and NAA.

The positive mass spectrum of alkaloid fractions in roots and leaves of ungrafted *Annona emarginata* and *Annona emarginata* grafted with *Annona atemoya* treated with auxins (IAA, IBA and NAA) showed ions with even *m*/*z* values between 200 and 400, suggesting the presence of protonated alkaloids in all samples, highlighting the most abundant ions (60–100% of relative abundance) 266, 276, 300, 306, 314, 328 and 330, and the less abundant ions (more than 5% of relative abundance) as possible alkaloids (Table 3). This proposal was corroborated by previous studies with alkaloids containing an odd number of nitrogen atoms, such as aporphine, proaporphine and oxoaporphine, where the protonation process provides products with even *m*/*z* values [34,35,36]. The following are among the tentatively identified alkaloids: anonaine (**1**), asimilobine (**2**), liriodenine (**3**), N-methylanonaine (**4**), nornuciferine (**5**), lysicamine (**6**), N-formyl-anonaine (**7**), xylopine (**8**), stepharine (**9**), 4′-*O*-methylcoclaurine (**10**), lanuginosine (oxoxylopine) (**11**), N,O-dimethylcoclaurine (**12**), 7-hydroxy-7-methyl-N-formyl-anonaine (**13**), nornantenine (**14**), boldine (**15**) stepholidine (**16**), reticuline (**17**), subsessiline (**18**) and xylopinine (**19**) (Table 3 and Figure 1).

The 19 alkaloids identified were present in root and leaf samples of ungrafted *A. emarginata* seedlings and those grafted with atemoya regardless of treatments and collection time (Table S1).

**Figure 2:**
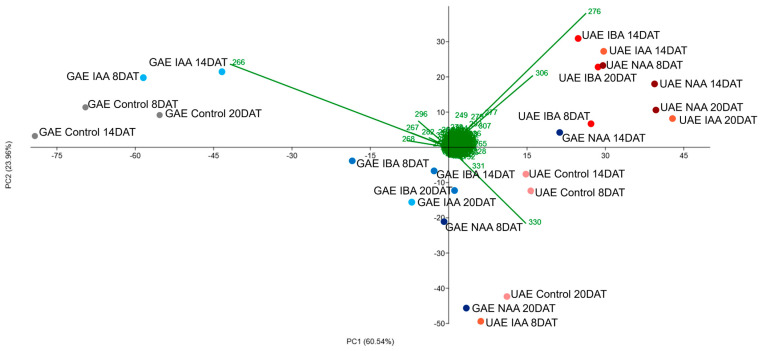
Principal component analysis (PCA) of alkaloids identified in the roots of ungrafted *Annona emarginata* (UAE) and *A. emarginata* grafted with *Annona atemoya* (GAE) treated with IAA, IBA and NAA in three collection times (8, 14 and 20 DAT) analyzed by APCI-MS.

**Figure 3:**
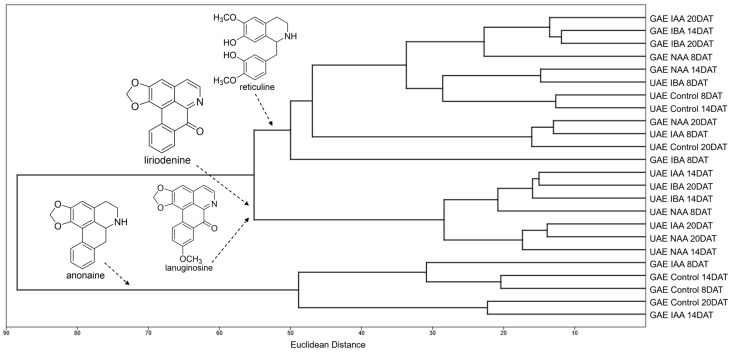
Hierarchical cluster analysis (HCA) of alkaloids identified in the roots of ungrafted *Annona emarginata* (UAE) and *A. emarginata* grafted with *Annona atemoya* (GAE) treated with IAA, IBA and NAA in three collection times (8, 14 and 20 DAT) analyzed by APCI-MS.

According to the principal component analysis (PCA) and hierarchical cluster analysis (HCA), the ions with the greatest intensity obtained in the DI-MS analysis (Figures S19–S34) were able to separate the roots and leaves of grafted and ungrafted plants by treatments. Anonaine (*m*/*z* 266) and asimilobine (*m*/*z* 268) ions were responsible for grouping the alkaloidal extracts of roots of *A. emarginata* seedlings grafted with *A. atemoya* from the control group at the three collection times and of seedlings treated with IAA at 8 and 14 DAT. Reticuline ion (*m*/*z* 330) grouped the roots of grafted seedlings treated with IBA in the three collection times, NAA at 8 and 20 DAT, and treated with IAA at 20 DAT (Figure 2 and Figure 3), and liriodenine (*m*/*z* 276) and lanuginosine ions (*m*/*z* 306) were responsible for the separation of roots of grafted seedlings treated with NAA at 14 DAT, which indicates a differential modulation of the three auxins in the biosynthesis of these alkaloids.

In the alkaloidal extracts of ungrafted *A. emarginata* roots, it is possible to observe that the ions with the highest intensity are different in the control group when compared to plants treated with auxins (IAA, IBA and NAA). PCA and HCA demonstrate that the control was grouped by the reticuline ion (*m*/*z* 330) in the three collection times, while seedlings treated with IBA and naphthalene acetic acid in the three collection times and IAA at 14 and 20 DAT are grouped by liriodenine (*m*/*z* 276) and lanuginosine ions (*m*/*z* 306) (Figure 2 and Figure 3).

When comparing the roots of ungrafted *A. emarginata* seedlings and seedlings grafted with *atemoya* from the control groups, the alkaloids with the highest intensity differ between the types of seedlings with the reticuline ion (*m*/*z* 330) having the highest intensity in the roots of ungrafted seedlings (Figure S19) and the anonaine ion (*m*/*z* 266) in roots of seedlings grafted with atemoya (Figure S23). This difference is indicative of the modulation caused by the grafting process, since the roots of both types of seedlings are of the same species (*A. emarginata*), and in grafted seedlings, the shoot is of *A. atemoya*.

**Figure 4:**
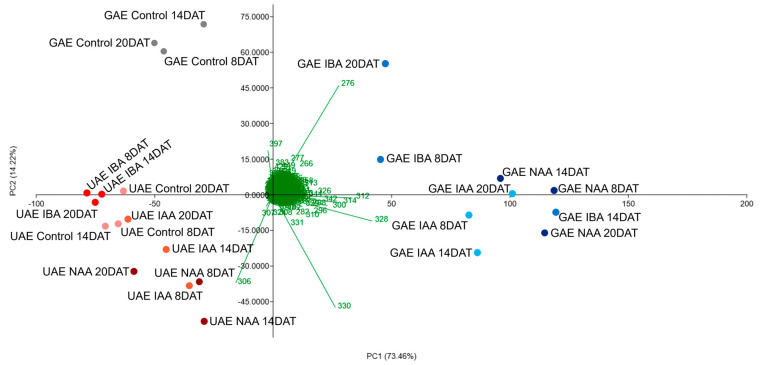
Principal component analysis (PCA) of alkaloids identified in the leaves of ungrafted *Annona emarginata* (UAE) and *Annona atemoya* grafted onto *Annona emarginata* (GAT) treated with IAA, IBA and NAA in three collection times (8, 14 and 20 DAT) analyzed by APCI-MS.

**Figure 5:**
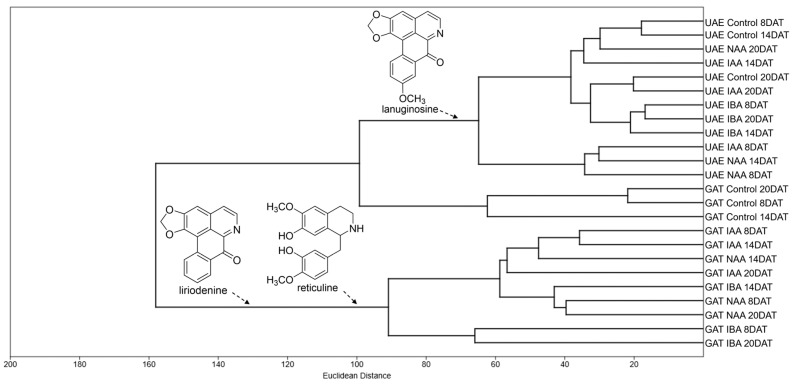
Hierarchical cluster analysis (HCA) of alkaloids identified in the leaves of ungrafted *Annona emarginata* (UAE) and *Annona atemoya* grafted onto *Annona emarginata* (GAT) treated with IAA, IBA and NAA at three collection times (8, 14 and 20 DAT) analyzed by APCI-MS.

In the leaves of *Annona atemoya* grafted onto *A. emarginata*, the effect of auxins on the modulation of alkaloids can be observed in PCA and HCA, where the control group in the three collection times differs from leaves treated with IAA, NAA and IBA in the three collection times, which are grouped by liriodenine (*m*/*z* 276), boldine (*m*/*z* 328), stepholidine (*m*/*z* 328) and reticuline ions (*m*/*z* 330) (Figure 4 and Figure 5).

On the other hand, the alkaloidal extracts in the leaves of *Annona emarginata* seedlings did not show any difference from the control with the species being grouped by the lanuginosine ion (*m*/*z* 306) (Figure 4 and Figure 5).

This behavior indicates a differential modulation of auxins depending on the species and the organ evaluated, since *A. emarginata* roots (ungrafted or used as rootstock) showed modulation in the intensity of ions detected in the three collection times and with the three auxins, while *A. emarginata* leaves showed no modulation.

Based on the analysis of the MS^2^ spectra of ions with the highest intensity, important previously described fragmentations were observed, which corroborate the identifications presented [34,35,36,39]. The fragmentation pattern of the ion at *m*/*z* 266 is consistent with alkaloid anonaine (**1**), as it presents an initial loss of 17 Da (-NH_3_) (*m*/*z* 266 → 249, Figure S1), which was followed by a loss of 30 Da (-CH_2_O) (*m*/*z* 249 → 219, Figure S1) and 28 Da (-CO) (*m*/*z* 219 → 191, Figure S1), providing strong evidence of an aporphine skeleton with a methylenedioxy group and the presence of the N-methyl group [35].

The ion 268 presents an initial loss of 17 Da (-NH_3_) (*m*/*z* 268 → 251, Figure S2), with subsequent loss of 32 Da (-CH_3_OH) (*m*/*z* 251 → 219, Figure S6), which indicates the presence of alkaloid asimilobine (**2**) [33,34,35]; the ion at *m*/*z* 276 is consistent with alkaloid liriodenine (**3**), presenting an intense fragmentation in relation to the loss of 28 Da (-CO) (*m*/*z* 276 → 248, Figure S3) [35,45].

The MS^2^ spectrum of the ion at *m*/*z* 330 showed a characteristic observed in benzylisoquinolinic alkaloids with high charge mass loss (*m*/*z* 330 → 192, Figure S16) and the base peak fragment at *m*/*z* 192, which is a key fragment commonly observed in N-methyl benzylisoquinoline skeletons containing methoxyl and hydroxyl substituents in ring A, such as alkaloid reticuline (**17**) (Figure S16) [35]. The ion at *m*/*z* 306 showed a single fragmentation in MS^2^ with a loss of 15 Da (-CH_3_) (*m*/*z* 306 → 291, Figure S11), which is consistent with the skeleton of the oxoaporphinic alkaloid lanuginosine (oxoxylopine) (**11**) [33,36].

In the analysis of the MS² spectra of the less abundant ions, it was possible to find key fragmentations that aid in the identification of alkaloids; ion 292 presents an initial loss of 15 Da (-CH_3_) (*m*/*z* 292 → 277, Figure S5) with a subsequent loss of 28 Da (-CO) (*m*/*z* 277 → 249, Figure S5), which indicates the presence of alkaloid lysicamine (**6**) [33,34,35].

Ions 280 and 296 exhibit initial losses of 31 Da (-NH_2_CH_3_) (*m*/*z* 280 → 249, Figure S4) and 17 Da (-NH_3_) (*m*/*z* 296 → 279, Figure S8), followed by losses of 30 Da (-CH_2_O) and 28 Da (-CO) (*m*/*z* 249 → 219 → 191, Figure S4 and *m*/*z* 279 → 249 → 221, Figure S8), which were indicative of alkaloids N-methylanonaine (**4**) and xylopine (**8**) [33,35].

The ions at *m*/*z* 314 and 338 showed characteristics of alkaloids N,O-dimethylcoclaurine (**12**) and subsessiline (**18**), respectively, with losses of 31 Da (-NH_2_CH_3_) (*m*/*z* 314 → 283, Figure S12) and 15 Da (-CH3) (*m*/*z* 338 → 323, Figure S18) [39,43].

Ions 282, 298, and 326 present characteristics indicative of alkaloids nornuciferine (**5**), stepharine (**9**), and nornantenine (**14**), respectively, showing an initial loss of 17 Da (-NH_3_) (*m*/*z* 282 → 265, Figure S5; *m*/*z* 298 → 281, Figure S9, and *m*/*z* 326 → 309, Figure S14) with subsequent losses of 15 Da (-CH_3_) (*m*/*z* 265 → 250, Figure S5; *m*/*z* 281 → 266, Figure S9, and *m*/*z* 309 → 294, Figure S14) and 31 Da (-NH_2_CH_3_) (*m*/*z* 265 → 234, Figure S5; *m*/*z* 281 → 250, Figure S9 and *m*/*z* 309 → 278, Figure S14) [33,34,35,39,42].

This work brings as a novelty the first report of the identification of alkaloids N-methylanonaine (**4**), N-formyl-anonaine (**7**), 4′-*O*-methylcoclaurine (**10**), N,O-dimethylcoclaurine (**12**), 7-hydroxy-7-methyl-N-formyl-anonaine (**13**), nornantenine (**14**), and subsessiline (**18**) in *A. emarginata* roots and leaves and in *A. atemoya* leaves as well as the first report of the presence of alkaloid stepharine (**9**) in *Annona emarginata* roots and leaves and of alkaloids xylopine (**8**) and xylopinine (**19**) in *A. atemoya* leaves.

## 3. Discussion

Auxins (IAA, IBA and ANA) significantly influence the synthesis of total alkaloids and the alkaloid profile of ungrafted *A. emarginata* seedlings and *A. emarginata* seedlings grafted with *A. atemoya*. This modulation showed differences depending on the type of auxin, species, grafting and the organ analyzed.

The increase in the concentration of total alkaloids in roots was caused by the supply of the three auxins in ungrafted *A. emarginata* and *A. emarginata* grafted with *A. atemoya* with a difference only in the response time (14 vs. 20 DAT, respectively). A similar result was found by Sousa et al. [20] only in the supply of IBA, which led to an increase in the synthesis of total alkaloids and liriodenine in the roots of ungrafted seedlings. This study shows the sensitivity of the metabolism of alkaloids in *A. emarginata* to auxin-type plant regulators. On the other hand, in leaves, the action of auxins was different, and in ungrafted *A. emarginata* seedlings, an increase was observed only with IBA (20 DAT), and in seedlings grafted with *A. atemoya*, the three auxins led to an increase in total alkaloids (8 and 20 DAT). It is important to remember that due to the grafting process, the shoots of seedlings are different; therefore, this finding indicates that the species respond differently and/or that the grafting process is more sensitive to auxins.

Regarding the effect of auxins on alkaloid production previously evaluated in *A. emarginata* “terra fria” by our research group [20,46], a particularity can be observed in relation to the response time. In Martin et al. [46], rapid responses to IAA were detected; and in leaves, there was a slight increase at 12 h, but the significant effect was observed at 324 h (13.5 DAT), while in roots at 36 and 156 h, the alkaloid production decreased, but at 13.5 days, the production was equal to control. In contrast, Sousa et al. [20] detected significant increases in roots at 20 DAT with the supply of IBA. This study highlights that the response of alkaloid metabolism to auxins in roots is more robust at later times than in the first hours of application, since the increase in the production of these nitrogen metabolites was detected from 8 DAT in roots and increased over time (14 and 20 DAT). It is also important to consider some differences in the methods used, which may be the reason for these differences. In Martin et al. [46], seedlings were almost two years old and had been growing for 10 months with nutrient supply. Furthermore, IAA was applied three times every 48 h and not twice in a period of seven days as in this study.

It was also observed that the yield of alkaloids in leaves was 10 to 20 times greater than in Martin et al. [46]. These differences in the volume of alkaloids can be attributed to natural variations and the conditions of each type of botanical material evaluated. As previously mentioned, in Martin et al. [46], seedlings were not only one year older but had also been growing with nutrient supplements for almost another year. It has been observed that some species, when they have optimal or superoptimal nutrients, tend to direct their resources to vegetative growth compared to plants under normal or stressed conditions that tend to invest more resources in defense molecules [47]. The amounts of root alkaloids in this study were also higher than those of Martin et al. [46] but by a smaller margin (between 1.5 and two times more), although the volume detected here was similar to that observed in control and *A. emarginata* seedlings treated with IBA by Sousa et al. [20] and to control and water-stressed seedlings by Honório et al. [32].

The observed modulation of alkaloid synthesis by auxins is likely due to their ability to regulate the expression of enzymes involved in the synthesis pathways of these metabolites. Alkaloid biosynthesis requires precursor molecules derived from primary metabolism, and their main synthesis pathway is that of shikimic acid. This pathway produces aromatic amino acids tryptophan, tyrosine and phenylalanine, which serve as essential precursors for alkaloid synthesis [48,49,50]. Studies have reported that the supply of auxins regulates the activity of the enzyme L-tryptophan decarboxylase, which is involved in the synthesis of tryptophan, with regulation depending on the source of auxin and the tissue under study [51]. This also occurs in the synthesis of tyrosine, a precursor of benzylisoquinolinic alkaloids (BIAs), such as those identified in this study. Koirala et al. [52] demonstrated that the supply of auxin 2,4-dichlorophenoxyacetic acid (2,4 D) + light in *Crinum x powellii* ‘Album’ (swamp lily) was able to increase the synthesis of tyrosine, which was reflected in the increase in alkaloids cherylline and lycorine. Other studies with cell suspension cultures corroborate the result on the increase in alkaloids by the exogenous supply of auxins [53,54,55].

The results of the alkaloid profile confirm this differential modulation. Similar to the concentration of total alkaloids, *A. emarginata* roots (ungrafted and rootstock) and *A. atemoya* (leaves) present alterations in the proportion of ions of greater intensity modulated by the different auxins. The identified ions belong to the class of benzylisoquinolinic alkaloids, and in particular to alkaloids aporphinic and oxoaporphinic, which are the most abundant alkaloids found in the Annonaceae family [8].

Although scarce, there are studies with other species demonstrating that the proportion and/or concentration of benzylisoquinolinic alkaloids (BIAs) can be altered by the supply of auxins. For example, the application of 5 µM of IAA from *Eschscholtzia californica* suspension cultures led to an increase in total alkaloids and BIA macarpine [55]. This type of cultivation also allows identifying that the effect of auxins depends on the species. *Thalictrum flavum* and *T. dipterocarpum* cultivation produces BIA berberine as the main alkaloid (0.3 and 0.4 g L^−1^, respectively). However, the production of berberine in *T. dipterocarpum* is stimulated by auxin NAA in combination with 6-benzylaminopurine, while in *T. flavum*, it was significantly suppressed by the same auxin [53]. Mekky et al. [56] demonstrated that the synthesis of indole alkaloids vincristine and vinblastine was also enhanced by the application of kinetin associated with IAA and reduced by the supply of Kinetin + 2,4D.

The alkaloids identified with greater intensity in both seedlings are of interest for the bioprospection of molecules, since they present biological activity [33,44,57,58], as is the case of anonaine (**1**), liriodenine (**3**), lanuginosine (**11**) and reticuline (**17**) identified in the roots of *A. emarginata* (ungrafted and rootstock) and in *A. atemoya* and *A. emarginata* leaves. Among the properties, activity against fungi is reported, highlighting the effect of the alkaloidal extract of *A. emarginata* against fungi that causes anthracnose (*Colletotrichum fructicola* and *Colletotrichum theobromicola*) [33] and the antifungal activity of liriodenine against *Rhizopus stolonifer* and *Aspergillus glaucus* [57]. Furthermore, the effect of liriodenin against *Candida* sp., *Cryptococcus neoformans* and *Cryptococcus gatii*, fungi that cause endemic systemic mycoses in humans, has also been reported [58], and the cytotoxic activity of lanuginosin in HepG2 cells (human hepatocellular carcinoma), presenting a 50% inhibitory concentration (IC50) below 4 μg/mL [44].

In addition, the increase in the synthesis and variation in the profile of antifungal alkaloids [33,57] caused by the supply of auxins may indicate an increase in the tolerance of these species to pathogen attacks, thus adding ecological and economic value to the species.

Another relevant aspect observed in relation to the synthesis of alkaloids in roots is due to the grafting process. Several studies have suggested that grafting may affect the synthesis of specialized metabolites [59,60,61]. The results observed in this study allow inferring that grafting promotes the modulation of the synthesis of total alkaloids, since the concentration of total alkaloids in *A. emarginata* roots (control) was naturally higher (approx. 40–66%) when it was used as a rootstock for *A. atemoya* (8 and 14 DAT), which characterizes the influence of the scion on the rootstock. In addition, it was observed that the major alkaloids are different in the roots of the control group of grafted *A. emarginata* (anonaine) and ungrafted *A. emarginata* (reticuline).

No studies on this topic have been found in literature; however, it has been documented that the degree of development and type of tissue are important for sensitivity to auxins, and they may therefore favor the production of certain alkaloids: for example, calluses, roots and bulbils of *Narcissus tazetta* produce Amaryllidaceae-type alkaloids (AAs) galantamine and lycorine when treated with auxins 2,4-D, 4-amino-3,5,6-trichloropicolinic acid (Picloram) and NAA, regardless of the concentration used, while AAs desmethylmaritidine and tazettine were found only in differentiated tissues when treated with 25 or 50 μM of NAA or in calluses with 50 μM of Picloram [62]. AAs, like ABIs, are alkaloids derived from tyrosine [63].

## 4. Materials and Methods

### 4.1. Plant Material

The plant material used, consisting of *Annona emarginata* (Schltdl.) H. Rainer seedlings, ‘terra-fria’ morphotype (ungrafted) and *A. emarginata*, ‘terra-fria’ morphotype grafted with *Annona atemoya* Mabb., was produced at the Seedling Production Center of CATI (Technical and Integral Assistance Coordination) in the municipality of São Bento do Sapucaí—São Paulo, Brazil. Branches of matrices were identified by a specialist (PhD. Jennifer Lopes, personal communication), who also supervised the dissection of the fertile material, observation of the macromorphology [64] and identification of the key of the genus *Annona* [65], and exsiccates were deposited at the “Irina Delanova Gemtchujnicov” plant complex (BOTU), Institute of Biosciences, UNESP, Campus de Botucatu—São Paulo, Brazil, under records No. 27600 (*Annona emarginata* (Schltdl.) H. Rainer ‘terra-fria’ morphotype and No. 28233 (*Annona atemoya* Mabb., Thompson var.).

Seedlings were produced by sowing *A. emarginata* in sand beds, and after emergence, seedlings were transplanted into black polyethylene bags containing commercial substrate based on pine bark (Carolina Soil^®^, Kingston, NC, USA). When they reached approximately six months of age, seedlings were divided into two batches. The first consisted of ungrafted seedlings, that is, seedlings originated from seeds, and the second batch was grafted with a clone of *A. atemoya* ‘Thompson’ cultivar (scion), using English grafting, according to standards routinely used in the CATI nursery.

After approximately 1.5 years, when seedlings reached an average height of 1.20 m, they were transferred to a greenhouse at the Department of Biodiversity and Biostatistics, Institute of Biosciences, UNESP, Botucatu Campus—São Paulo, Brazil (located at 22°53′25″ S, 48°27′19″ W, and altitude of 800 m a.s.l.; Cwa climate). After 60 days of acclimatization, plants were submitted to treatments with auxins.

### 4.2. Experimental Design

The experiment was conducted in a completely randomized experimental design with a 2 × 4 factorial scheme with 2 types of seedlings (ungrafted *Annona emarginata* and *Annona emarginata* grafted with *Annona atemoya*) and four treatments (control, IAA, IBA and NAA). Evaluations were carried out three times after the start of treatments (8 DAT, 14 DAT and 20 DAT).

### 4.3. Application of Plant Growth Regulators

Two foliar applications of treatments (auxins) were carried out with an interval of seven days between them. IAA, IBA and NAA (Sigma-Aldrich^®^ Brasil Ltda., São Paulo, Brazil) at a concentration of 10^−6^ M were used as sources of auxin [20]. Plant regulators were solubilized in deionized water and ethyl alcohol, and 0.01% of Haiten^®^ non-ionic adhesive (Ningbo, China) was added to the solution, according to the manufacturers’ recommendations.

IAA, IBA and NAA applications were performed using a manual pressurized carbon dioxide sprayer with a fan nozzle at flow rates of 0.09 to 9.464 L/min and pressures of up to 276 bar [46].

### 4.4. Quantitative and Qualitative Analyses of Alkaloids

#### 4.4.1. Obtaining Total Alkaloid Extracts and Quantification

To obtain total alkaloid extracts, 10 g of roots and leaves was collected per replicate. The plant material was dried in a forced aeration oven at 30 °C until constant dry matter was obtained, and then extraction was performed using the acid–base method [66]. The total alkaloid content was determined by a UV-Vis spectrophotometer (model UV-M51—BEL Engineering^®^, Monza, Italy) at 254 nm, using the alkaloid liriodenine to elaborate the standard curve (y = 0.0881x − 0.0112, R^2^ = 0.9949; LOD = 0.445 μg mL^−1^ and LOQ = 1.205 μg mL^−1^), since it is a major alkaloid in *A. emarginata* [67].

#### 4.4.2. Analysis of the Alkaloid Profile by DI-MS

The stock solutions of alkaloid fractions were prepared at 1 mg mL^−1^ in methanol and were subsequently diluted to 10 μg mL^−1^, being analyzed by direct infusion in the LCQ Fleet mass spectrometer (Thermo Scientific, San Jose, CA, USA) equipped with an atmospheric pressure chemical ionization source—APCI and operating in positive ion mode for MS and MS/MS analyses [35,37]. Spectra were obtained by averaging at least 10 acquired spectra representing the pooled triplicate of each sample. The analytical conditions for the determinations were discharge current, 5 µA; vaporizer temperature, 350 °C; sheath gas pressure, 35 arbitrary unit (arb); ion sweep gas pressure, 0.0 arb; auxiliary gas pressure, 15 arb; capillary temperature, 250 °C; tube lens offset, 112 V; skimmer offset, 0 V; mass range, *m*/*z* 200 to 400; collision gas, He. MS/MS spectra were obtained using collision energy ranging from 20% to 30%.

### 4.5. Data Analysis

Total alkaloid data were submitted to analysis of variance (two-way ANOVA) and the means were compared by Tukey’s test at a significance level of 5% (*p* < 0.05) on each collection date.

Multivariate analysis of data from DI-MS analyses was performed using the Past 4.17 software [68]. Principal component analysis (PCA) and hierarchical cluster analysis (HCA) were calculated using the Euclidean distance, and the mean binding of the variation in ions was recorded between *m*/*z* 150 and 400 (variables). Cluster analysis was applied to compare groups based on treatments (auxin) and type of seedlings (grafted and ungrafted).

## 5. Conclusions

Auxins are plant regulators that modulate alkaloid production in *Annona emarginata* both in plants from seeds (ungrafted) and in seedlings grafted with *A. atemoya*. IAA, IBA, and NAA stimulate an increase in the total production of these specialized metabolites and modulate specific molecules in the chemical profile of roots and leaves, characterizing effects between scion and rootstock in the synthesis of alkaloids.

Among the 19 identified alkaloids, this study reports the presence of eight alkaloids in *A. emarginata* for the first time (N-methylanoneine (**4**), N-formyl-anoneine (**7**), stepharine (**9**), 4′-*O*-methylcoclaurine (**10**), N,O-dimethylcoclaurine (**12**), 7-hydroxy-7-methyl-N-formyl-anoneine (**13**), nornantenine (**14**), and subsessiline (**18**)). In addition to all the aforementioned alkaloids (except for stepharine), xylopine (**8**) and xylopinine (**19**) were also identified for the first time in the leaves of *A. atemoya*.

## Figures and Tables

**Table 1:** Concentration of total alkaloids (µg g^−1^ dry mass) in ungrafted *Annona emarginata* roots and grafted with *Annona atemoya* submitted to treatment with indoleacetic acid (IAA), indole butyric acid (IBA) and naphthalene acetic acid (NAA) at three collection times (8, 14 and 20 days after treatments—DAT).

Total Alkaloids in Roots (µg g^−1^ Dry Mass)		
8 DAT		
	Ungrafted *A. emarginata*	Grafted *A. emarginata*
Control	201.322 ± 6.72 Bb	287.144 ± 40.5 BCa ^1^
IAA	297.661 ± 8.47 Aa	230.714 ± 3.51 Cb
IBA	197.750 ± 12.05 Bb	370.268 ± 7.2 Aa
NAA	310.033 ± 17.25 Aa	344.812 ± 7.06 ABa
Treatment *p* < 0.001; F: 8.74 Species *p* < 0.001; F: 22.10 Treatment × Species *p* < 0.001; F: 17.30		
14 DAT		
	Ungrafted *A. emarginata*	Grafted *A. emarginata*
Control	138.432 ± 15.16 Cb	230.212 ± 5.95 Ba
IAA	277.114 ± 18.83 ABa	272.171 ± 18.35 ABa
IBA	230.310 ± 7.39 Bb	332.696 ± 43.96 Aa
NAA	389.102 ± 42.53 Aa	236.860 ± 10.57 ABb
Treatment *p* < 0.001; F: 13.38 Species *p*: 0.260; F: 1.31 Treatment × Species *p* < 0.001; F: 11.12		
20 DAT		
	Ungrafted *A. emarginata*	Grafted *A. emarginata*
Control	185.863 ± 7.37 Ca	226.580 ± 4.71 Ba
IAA	311.020 ± 19.48 ABb	378.382 ± 36.37 Aa
IBA	245.452 ± 15.86 BCb	330.315 ± 19.72 Aa
NAA	380.146 ± 24.82 Aa	364.690 ± 14.42 Aa
Treatment *p* < 0.001; F: 26.55 Species *p*: 0.003; F: 9.71 Treatment × Species *p*: 0.089; F: 2.37		

^1^ Means ± standard error followed by the same letter, capital letter comparing treatments (column) and lowercase letter comparing species (row), do not differ by Tukey’s test at 5% probability.

**Table 2:** Concentration of total alkaloids (µg g^−1^ dry mass) in ungrafted *Annona emarginata* leaves and atemoya leaves (grafted plants) submitted to treatment with IAA, IBA and NAA at three collection times (8, 14 and 20 DAT).

Total Alkaloids in Leaves (µg g^−^¹ Dry Mass)		
8 DAT		
	Ungrafted *A. emarginata*	Grafted *A. atemoya*
Control	32.487 ± 1.30 ABa	20.511 ± 0.17 Db ^1^
IAA	34.572 ± 0.57 Ab	49.509 ± 2.38 Aa
IBA	29.168 ± 0.51 Ba	26.486 ± 2.11 Ca
NAA	35.963 ± 0.24 Aa	33.535 ± 1.94 Ba
Treatment *p* < 0.001; F: 50.05 Species *p*: 0.140; F: 2.29 Treatment × Species *p* < 0.001; F: 24.58		
14 DAT		
	Ungrafted *A. emarginata*	Grafted *A. atemoya*
Control	42.428 ± 0.94 Aa	43.996 ± 1.25 Ca
IAA	46.812 ± 1.03 Ab	66.666 ± 2.31 Aa
IBA	27.310 ± 1.90 Bb	51.495 ± 0.60 Ba
NAA	42.302 ± 0.81 Ab	47.579 ± 4.20 BCa
Treatment *p* < 0.001; F: 20.40 Species *p* < 0.001; F: 77.48 Treatment × Species *p* < 0.001; F: 11.49		
20 DAT		
	Ungrafted *A. emarginata*	Grafted *A. atemoya*
Control	33.079 ± 0.59 Ba	23.237 ± 3.36 Cb
IAA	45.663 ± 0.23 Aa	46.039 ± 2.58 Aa
IBA	24.938 ± 0.39 Cb	35.293 ± 0.74 Ba
NAA	34.236 ± 2.33 Ba	34.847 ± 1.44 Ba
Treatment *p* < 0.001; F: 37.55 Species *p*: 0.773; F: 0.08 Treatment × Species *p* < 0.001; F: 10.18		

^1^ Means ± standard error followed by the same letter, capital letter comparing treatments (column) and lowercase letters comparing species (row), do not differ by Tukey’s test at 5% probability.

**Table 3:** Alkaloids identified in ungrafted *Annona emarginata* and *Annona emarginata* grafted with *Annona atemoya*.

Alkaloid	[M+H]^+^ (*m*/*z*)	MS/MS (*m*/*z*)	Class *	References	Figures **
Anonaine (**1**)	266	249, 219, 191	A	[33,35]	Figure S1
Asimilobine (**2**)	268	251, 219	A	[33,34,35]	Figure S2
Liriodenine (**3**)	276	248, 220	O	[33,35,36,37]	Figure S3
*N*-methylanonaine (**4**)	280	249, 219, 191	A	[35]	Figure S4
Nornuciferine (**5**)	282	265, 250, 234	A	[33,34,35]	Figure S5
Lysicamine (**6**)	292	277, 249	O	[35]	Figure S6
*N*-formyl-anonaine (**7**)	294	249, 219, 191	A	[38]	Figure S7
Xylopine (**8**)	296	279, 249, 221	A	[33,35]	Figure S8
Stepharine (**9**)	298	281, 266, 250	P	[39]	Figure S9
4′-*O*-methylcoclaurine (**10**)	300	283	B	[40,41]	Figure S10
Lanuginosine (oxoxylopine) (**11**)	306	291	O	[33,36]	Figure S11
N,O-dimethylcoclaurine (**12**)	314	283	B	[39]	Figure S12
7-hydroxy-7-methyl-N-formyl-anonaine (**13**)	324	279, 249, 221	A	-	Figure S13
Nornantenine (**14**)	326	309, 294, 278	A	[42]	Figure S14
Boldine (**15**)	328	297, 265	A	[37]	Figure S15
Stepholidine (**16**)	328	297, 265	T	[35]	Figure S15
Reticuline (**17**)	330	192	B	[33,35]	Figure S16
Subsessiline (**18**)	338	323	O	[39,43]	Figure S17
Xylopinine (**19**)	356	192, 165	A	[44]	Figure S18

* Aporphine (A); oxoaporphine (O); proaporphine (P); benzylisoquinoline (B); tetrahydroberberine (T). ** APCI-MS^n^ spectrum figures presented in the Supplementary Materials.

## Data Availability

Data are contained in the article and Supplementary Materials.

## Supplementary Materials

The following supporting information can be downloaded at https://www.mdpi.com/article/10.3390/molecules30092070/s1, Table S1. Profile of identified alkaloids present in roots and leaves of ungrafted *Annona emarginata* and *A. emarginata* grafted with *Annona atemoya* treated with indole acetic acid (IAA), indole butyric acid (IBA) and naphthalene acetic acid (NAA); Figure S1. APCI-MS^n^ spectrum (positive mode) of ion *m*/*z* 266 (Anonaine); Figure S2. APCI-MS^n^ spectrum (positive mode) of ion *m*/*z* 268 (Asimilobine); Figure S3. APCI-MS^n^ spectrum (positive mode) of ion *m*/*z* 276 (liriodenine); Figure S4. APCI-MS^n^ spectrum (positive mode) of ion *m*/*z* 280 (*N*-methylanonaine); Figure S5. APCI-MS^n^ spectrum (positive mode) of ion *m*/*z* 282 (nornuciferine); Figure S6. APCI-MS^n^ spectrum (positive mode) of ion *m*/*z* 292 (lysicamine); Figure S7. APCI-MS^n^ spectrum (positive mode) of ion *m*/*z* 294 (*N*-formyl-anonaine); Figure S8. APCI-MS^n^ spectrum (positive mode) of ion *m*/*z* 296 (xylopine); Figure S9. APCI-MS^n^ spectrum (positive mode) of ion *m*/*z* 298 (stepharine); Figure S10. APCI-MS^n^ spectrum (positive mode) of ion *m*/*z* 300 (4′-*O*-methylcoclaurine); Figure S11. APCI-MS^n^ spectrum (positive mode) of ion *m*/*z* 306 (lanuginosine or oxoxylopine); Figure S12. APCI-MS^n^ spectrum (positive mode) of ion *m*/*z* 314 (N,O-dimethylcoclaurine); Figure S13. APCI-MS^n^ spectrum (positive mode) of ion *m*/*z* 324 (7-hydroxy-7-methyl-N-formyl-anonaine); Figure S14. APCI-MS^n^ spectrum (positive mode) of ion *m*/*z* 326 (nornantenine); Figure S15. APCI-MS^n^ spectrum (positive mode) of ion *m*/*z* 328 (boldine and stepholidine); Figure S16. APCI-MS^n^ spectrum (positive mode) of ion *m*/*z* 330 (reticuline); Figure S17. APCI-MS^n^ spectrum (positive mode) of ion *m*/*z* 338 (subsessiline); Figure S18. APCI-MS^n^ spectrum (positive mode) of ion *m*/*z* 356 (xylopinine); Figure S19. APCI-MS^n^ spectrum (positive mode) of total alkaloid extract from the root of ungrafted *Annona emarginata* without auxin supply (control) at 8 (A), 14 (B) and 20 (C) days after the beginning of the treatments (DAT); Figure S20. APCI-MS^n^ spectrum (positive mode) of total alkaloid extract from the root of ungrafted *Annona emarginata* treated with IAA at 8 (A), 14 (B) and 20 (C) DAT; Figure S21. APCI-MS^n^ spectrum (positive mode) of total alkaloid extract from the root of ungrafted *Annona emarginata* treated with IBA at 8 (A), 14 (B) and 20 (C) DAT; Figure S22. APCI-MS^n^ spectrum (positive mode) of total alkaloid extract from the root of ungrafted *Annona emarginata* treated with NAA at 8 (A), 14 (B) and 20 (C) DAT; Figure S23. APCI-MS^n^ spectrum (positive mode) of total alkaloid extract from the root of *Annona emarginata* grafted with *Annona atemoya* without auxin supply (control) at 8 (A), 14 (B) and 20 (C) DAT; Figure S24. APCI-MS^n^ spectrum (positive mode) of total alkaloid extract from the root of *Annona emarginata* grafted with *Annona atemoya* treated with IAA at 8 (A), 14 (B) and 20 (C) DAT; Figure S25. APCI-MS^n^ spectrum (positive mode) of total alkaloid extract from the root of *Annona emarginata* grafted with *Annona atemoya* treated with IBA at 8 (A), 14 (B) and 20 (C) DAT; Figure S26. APCI-MS^n^ spectrum (positive mode) of total alkaloid extract from the root of *Annona emarginata* grafted with *Annona atemoya* treated with NAA at 8 (A), 14 (B) and 20 (C) DAT; Figure S27. APCI-MS^n^ spectrum (positive mode) of total alkaloid extract from the leaves of ungrafted *Annona emarginata* without auxin supply (control) at 8 (A), 14 (B) and 20 (C) DAT; Figure S28. APCI-MS^n^ spectrum (positive mode) of total alkaloid extract from the leaves of ungrafted *Annona emarginata* treated with IAA at 8 (A), 14 (B) and 20 (C) DAT; Figure S29. APCI-MS^n^ spectrum (positive mode) of total alkaloid extract from the leaves of ungrafted *Annona emarginata* treated with IBA at 8 (A), 14 (B) and 20 (C) DAT; Figure S30. APCI-MS^n^ spectrum (positive mode) of total alkaloid extract from the leaves of ungrafted *Annona emarginata* treated with NAA at 8 (A), 14 (B) and 20 (C) DAT; Figure S31. APCI-MS^n^ spectrum (positive mode) of total alkaloid extract from the leaves of *Annona atemoya* (grafted plants) without auxin supply (control) at 8 (A), 14 (B) and 20 (C) DAT; Figure S32. APCI-MS^n^ spectrum (positive mode) of total alkaloid extract from the leaves of *Annona atemoya* (grafted plants) treated with IAA at 8 (A), 14 (B) and 20 (C) DAT; Figure S33. APCI-MS^n^ spectrum (positive mode) of total alkaloid extract from the leaves of *Annona atemoya* (grafted plants) treated with IBA at 8 (A), 14 (B) and 20 (C) DAT; Figure S34. APCI-MS^n^ spectrum (positive mode) of total alkaloid extract from the leaves of *Annona atemoya* (grafted plants) treated with NAA at 8 (A), 14 (B) and 20 (C) DAT. Figure S35. (A) Principal component analysis (PCA) and (B) hierarchical cluster analysis (HCA) of alkaloids identified in *Annona emarginata* roots grafted with *Annona atemoya* treated with IAA, IBA and NAA in three collection times (8, 14 and 20 DAT) analyzed by APCI-MS. Figure S36. (A) Principal component analysis (PCA) and (B) hierarchical cluster analysis (HCA) of alkaloids identified in the roots of ungrafted *Annona emarginata* submitted to treatments with IAA, IBA and NAA at three collection times (8, 14 and 20 DAT) analyzed by APCI-MS.

## Abbreviations

The following abbreviations are used in this manuscript:

IAA	indoleacetic acid
IBA	indolebutyric acid
NAA	naphthaleneacetic acid
DAT	days after treatments
APCI	atmospheric pressure chemical ionization
2,4 D	2,4-dichlorophenoxyacetic acid
BIAs	benzylisoquinolinic alkaloids
AAs	amaryllidaceae type alkaloids
PCA	principal component analysis
HCA	hierarchical cluster analysis
