# Supplementary material for: Effect of Auxins on the Accumulation of Alkaloids in Ungrafted Annona emarginata (Schltdl.) H. Rainer and Annona emarginata (Schltdl.) H. Rainer Grafted with Annona atemoya Mabb
Source: Molecules. 2025 May 7;30(9):2070. doi: 10.3390/molecules30092070 (PMC12073484; doi:10.3390/molecules30092070)
Supplement: Supplementary file 1 [file molecules-30-02070-s001.zip › molecules-3589954-supplementary.pdf]

Supplementary Materials

Table S1. Profile of identified alkaloids present in roots and leaves of ungrafted *Annona emarginata* and *A. emarginata* grafted with *Annona atemoya* treated with indole acetic acid (IAA), indole butyric acid (IBA) and naphthalene acetic acid (NAA).

	Ungrafted *Annona emarginata* (Roots/Leaves)				*Annona emarginata* grafted with *A. atemoya* (Roots/Leaves)			
	Control	IAA	IBA	NAA	Control	IAA	IBA	NAA
Anonaine (**1**)	x/x	x/x	x/x	x/x	x/x	x/x	x/x	x/x
Asimilobine (**2**)	x/x	x/x	x/x	x/x	x/x	x/x	x/x	x/x
Liriodenine (**3**)	x/x	x/x	x/x	x/x	x/x	x/x	x/x	x/x
N-methylanonaine (**4**)	x/x	x/x	x/x	x/x	x/x	x/x	x/x	x/x
Nornuciferine (**5**)	x/x	x/x	x/x	x/x	x/x	x/x	x/x	x/x
Lysicamine (**6**)	x/x	x/x	x/x	x/x	x/x	x/x	x/x	x/x
N-formyl-anonaina (**7**)	x/x	x/x	x/x	x/x	x/x	x/x	x/x	x/x
Xylopine (**8**)	x/x	x/x	x/x	x/x	x/x	x/x	x/x	x/x
Stepharine (**9**)	x/x	x/x	x/x	x/x	x/x	x/x	x/x	x/x
4’-O-methylcoclaurine (**10**)	x/x	x/x	x/x	x/x	x/x	x/x	x/x	x/x
Lanuginosine (oxoxylopine) (**11**)	x/x	x/x	x/x	x/x	x/x	x/x	x/x	x/x
N,O-dimethylcoclaurine (**12**)	x/x	x/x	x/x	x/x	x/x	x/x	x/x	x/x
7-hydroxy-7-methyl-N-formyl-anonaine (**13**)	x/x	x/x	x/x	x/x	x/x	x/x	x/x	x/x
Nornantenine (**14**)	x/x	x/x	x/x	x/x	x/x	x/x	x/x	x/x
Boldine (**15**)	x/x	x/x	x/x	x/x	x/x	x/x	x/x	x/x
Stepholidine (**16**)	x/x	x/x	x/x	x/x	x/x	x/x	x/x	x/x
Reticuline (**17**)	x/x	x/x	x/x	x/x	x/x	x/x	x/x	x/x
Subsessiline (**18**)	x/x	x/x	x/x	x/x	x/x	x/x	x/x	x/x
Xylopinine (**19**)	x/x	x/x	x/x	x/x	x/x	x/x	x/x	x/x

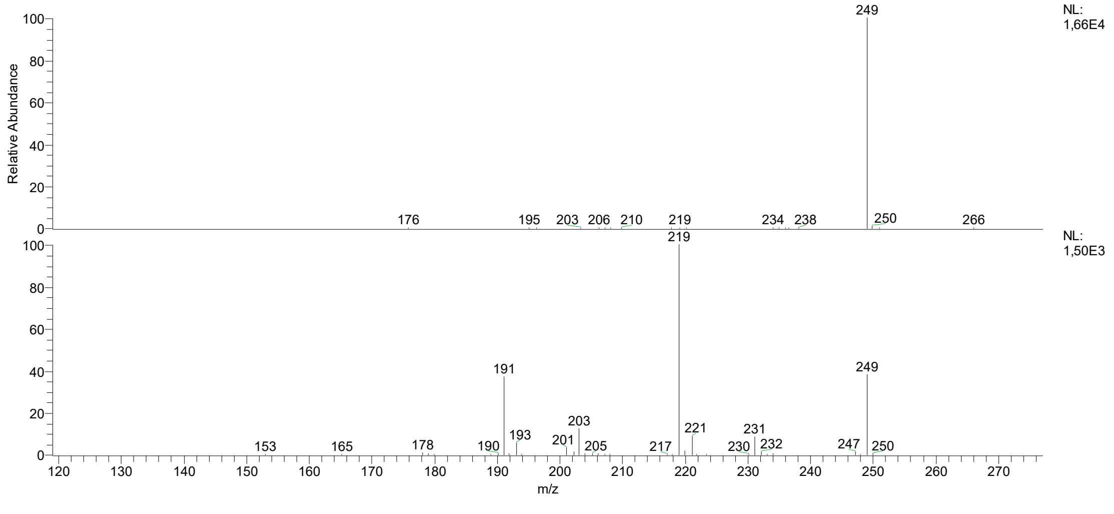

Figure S1. APCI-MS^n spectrum (positive mode) of ion *m/z* 266 (Anonaine).

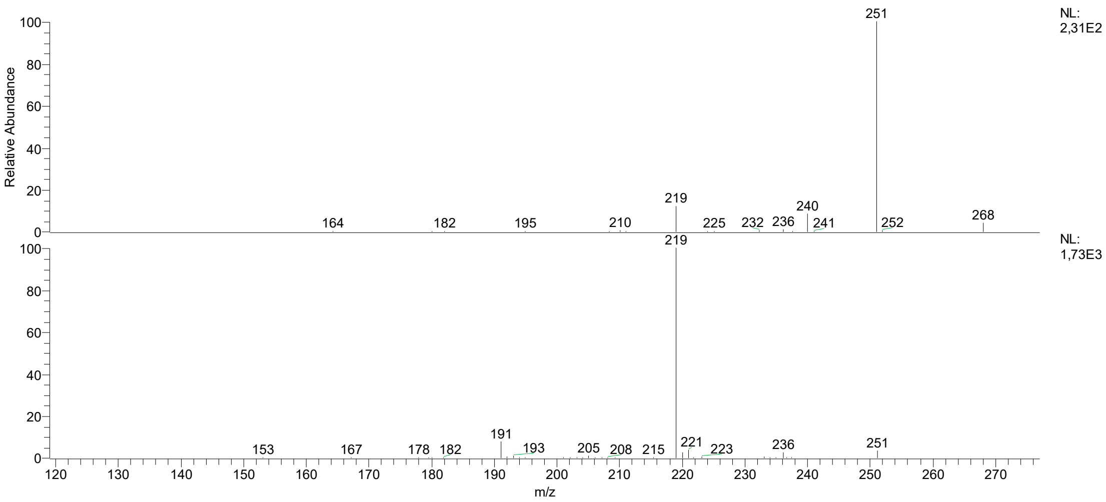

Figure S2. APCI-MS^n spectrum (positive mode) of ion *m/z* 268 (Asimilobine).

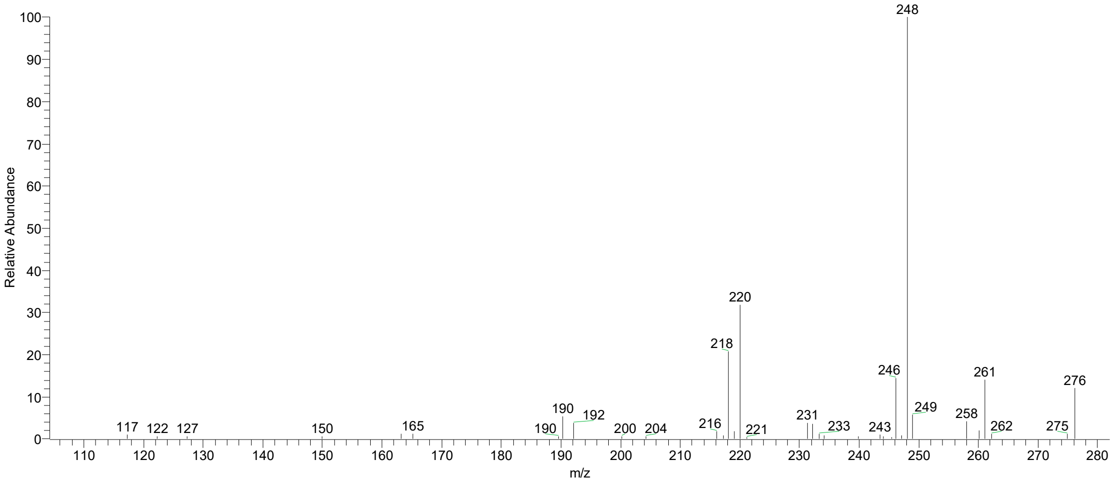

Figure S3. APCI-MS^n spectrum (positive mode) of ion *m/z* 276 (Liriodenine).

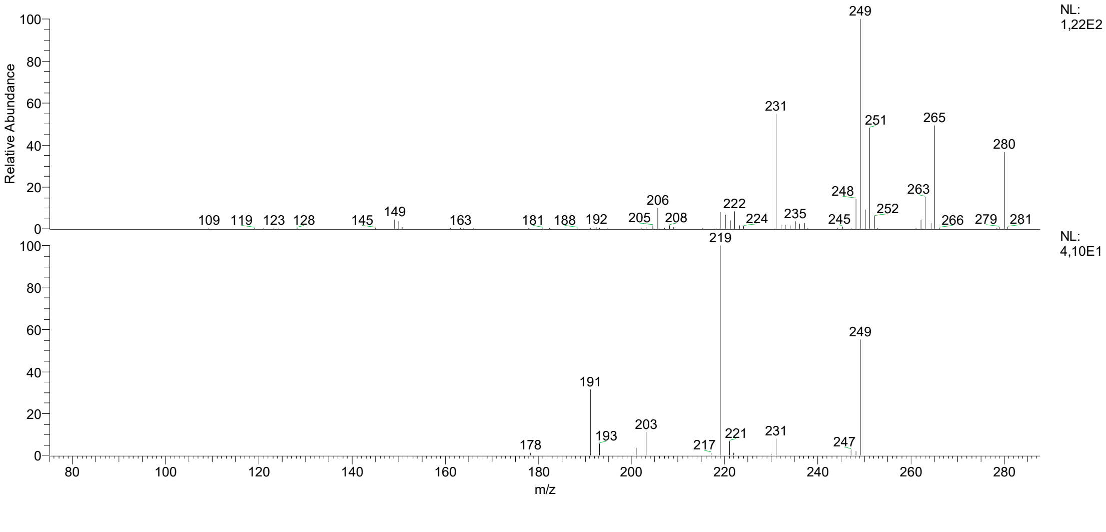

Figure S4. APCI-MS^n spectrum (positive mode) of ion *m/z* 280 (*N*-methylanonaine).

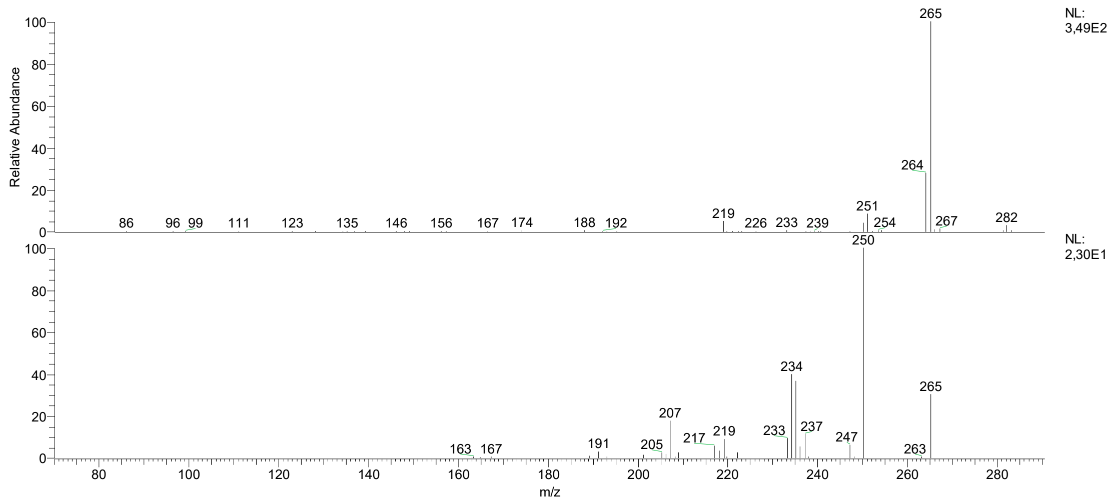

Figure S5. APCI-MS^n spectrum (positive mode) of ion *m/z* 282 (Nornuciferine).

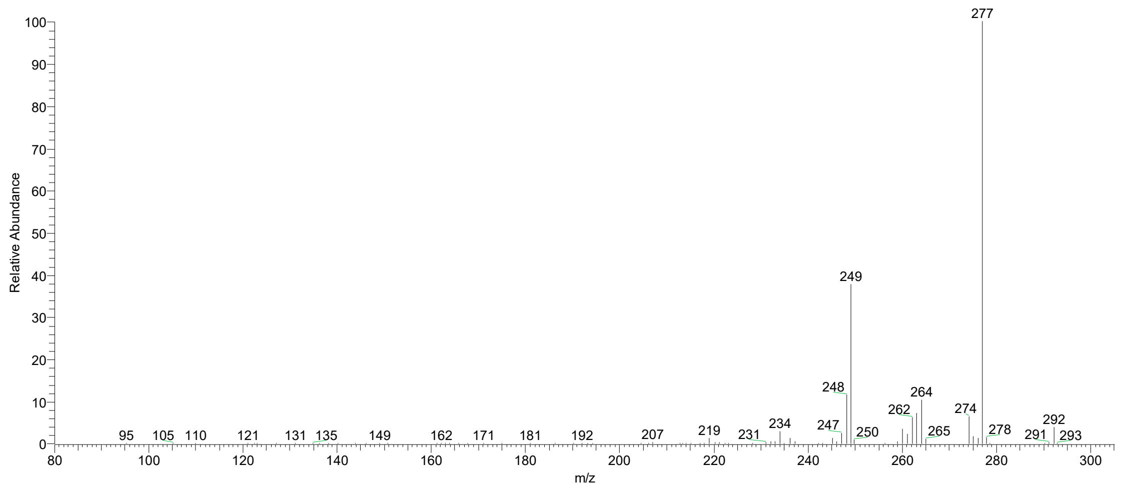

Figure S6. APCI-MS^n spectrum (positive mode) of ion *m/z* 292 (Lysicamine).

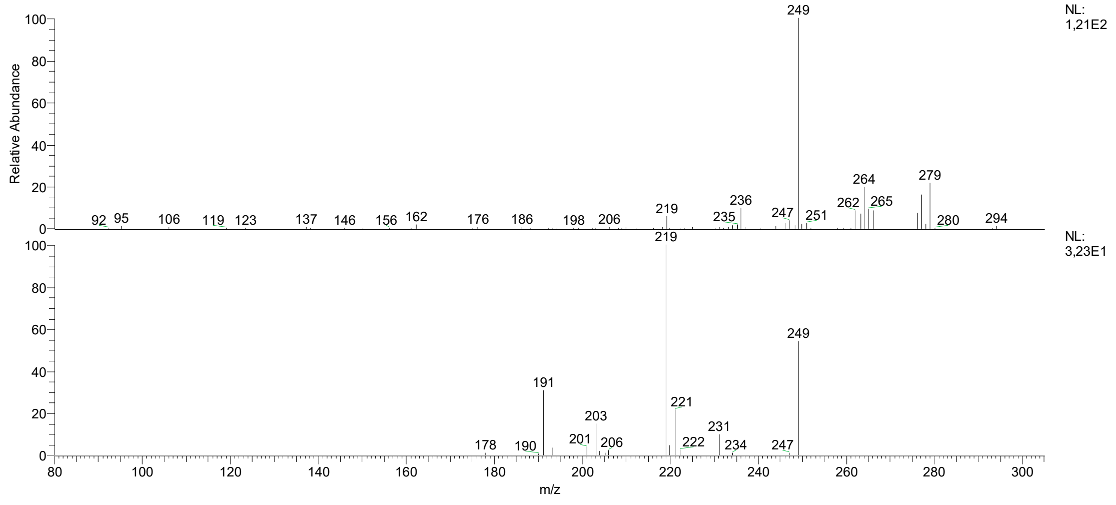

Figure S7. APCI-MS^n spectrum (positive mode) of ion *m/z* 294 (*N*-formyl-anonaine).

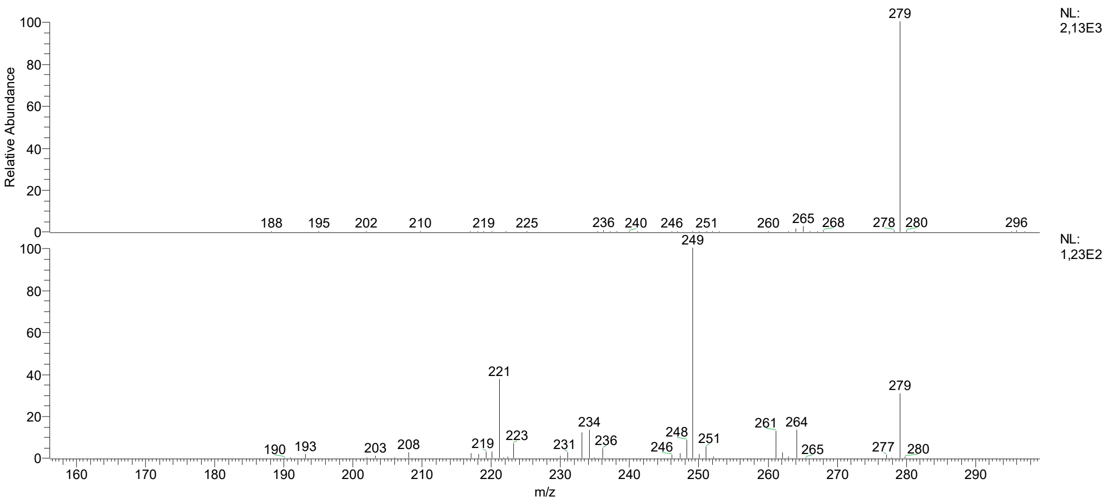

Figure S8. APCI-MS^n spectrum (positive mode) of ion *m/z* 296 (xylopine).

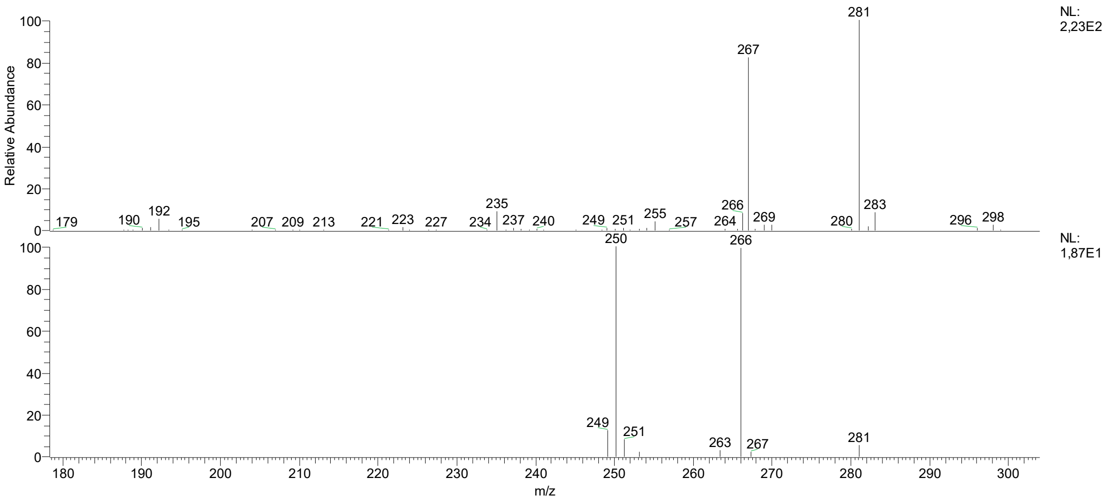

Figure S9. APCI-MS^n spectrum (positive mode) of ion *m/z* 298 (stepharine).

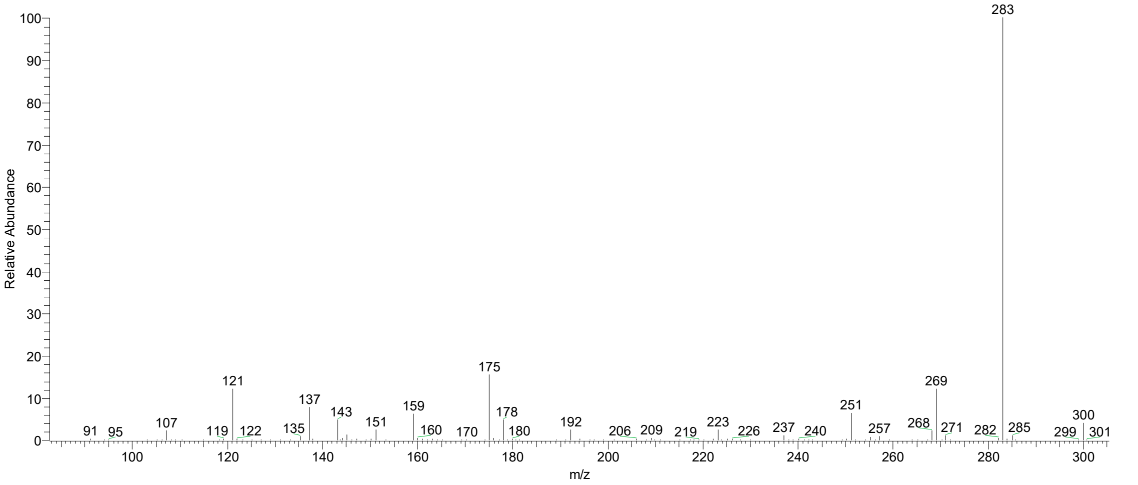

Figure S10. APCI-MS^n spectrum (positive mode) of ion *m/z* 300 (4'-O-methylcoclaurine).

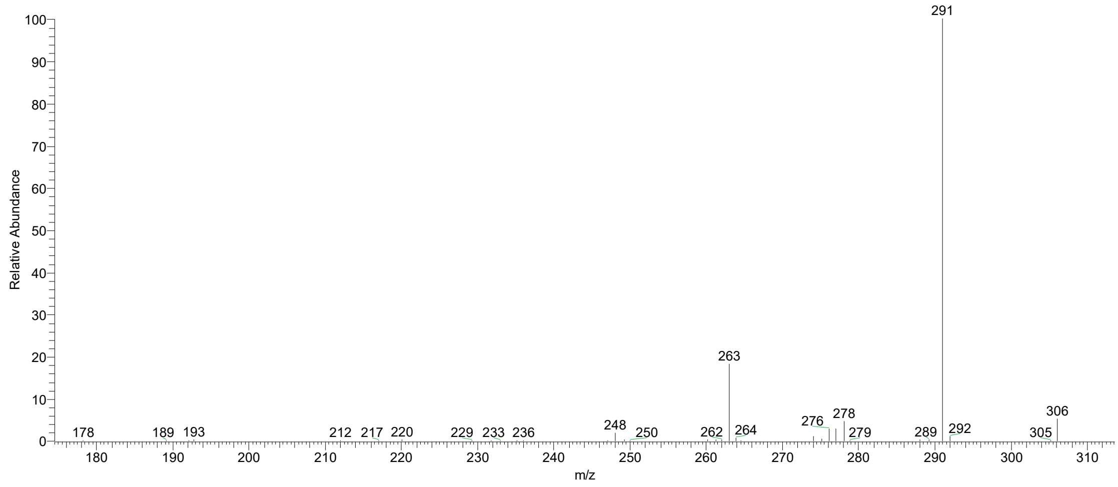

Figure S11. APCI-MS^n spectrum (positive mode) of ion *m/z* 306 (lanuginosine or oxoxylopine).

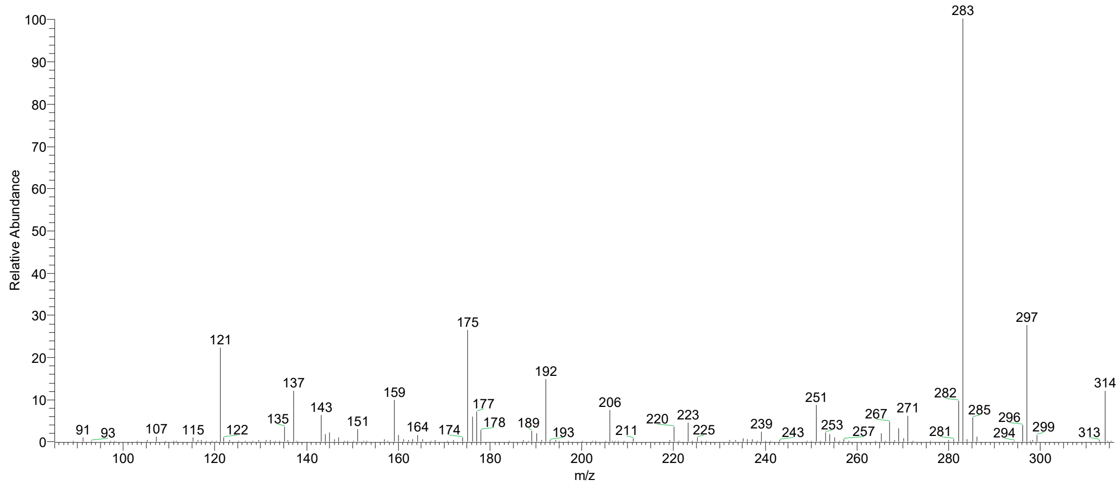

Figure S12. APCI-MS^n spectrum (positive mode) of ion *m/z* 314 (N,O-dimethylcoclaurine).

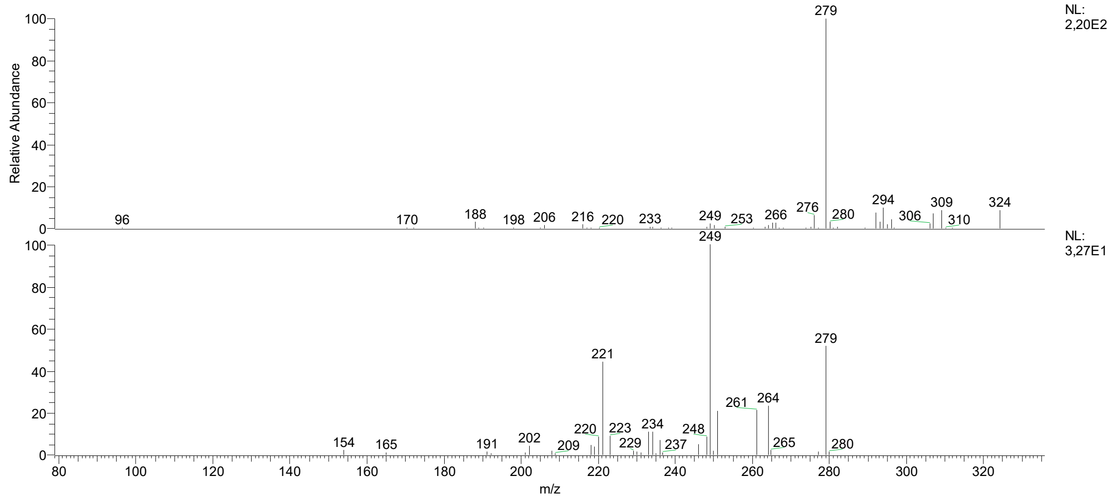

Figure S13. APCI-MS^n spectrum (positive mode) of ion *m/z* 324 (7-hydroxy-7-methyl-N-formyl-anonaine).

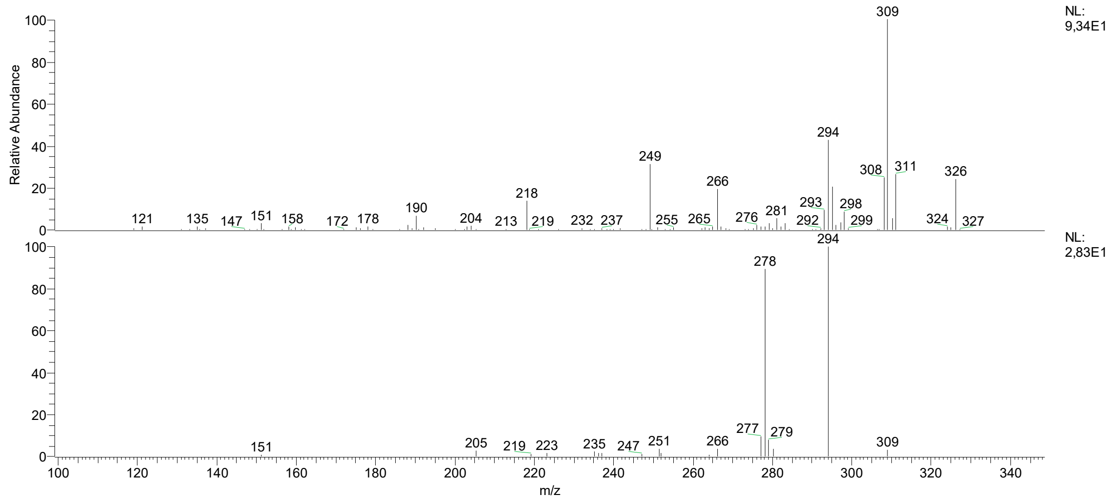

Figure S14. APCI-MS^n spectrum (positive mode) of ion *m/z* 326 (Nornantenine).

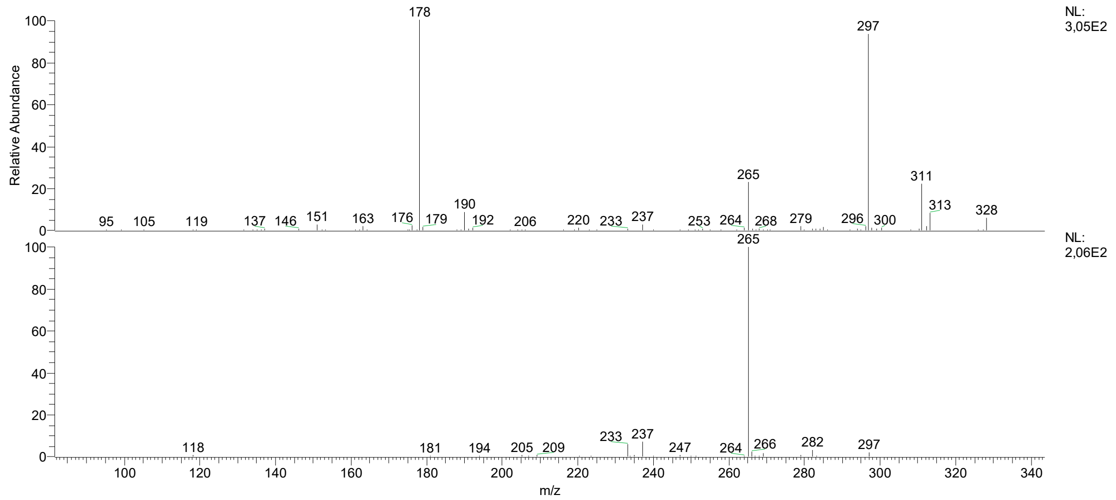

Figure S15. APCI-MS^n spectrum (positive mode) of ion *m/z* 328 (boldine and stepholidine).

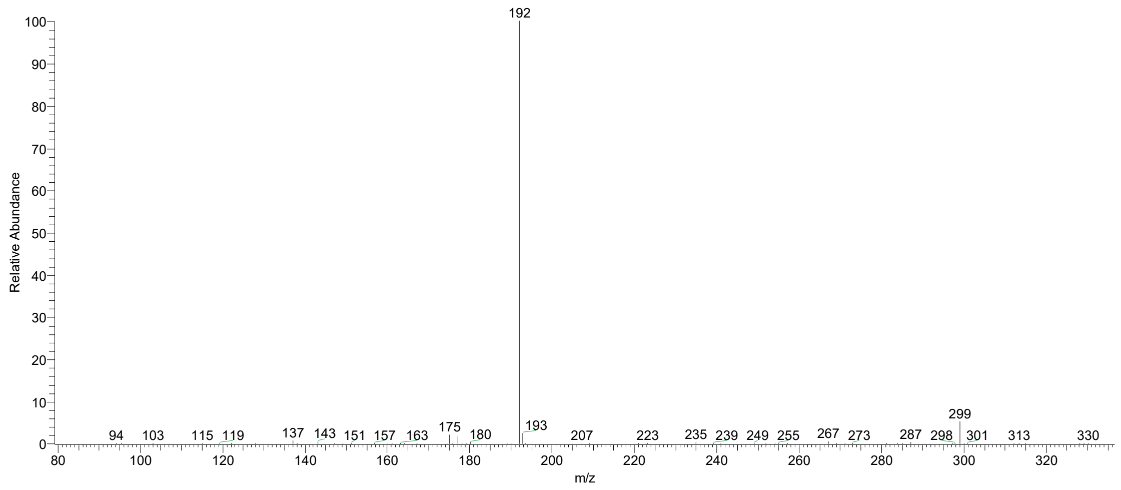

Figure S16. APCI-MS^n spectrum (positive mode) of ion *m/z* 330 (Reticuline).

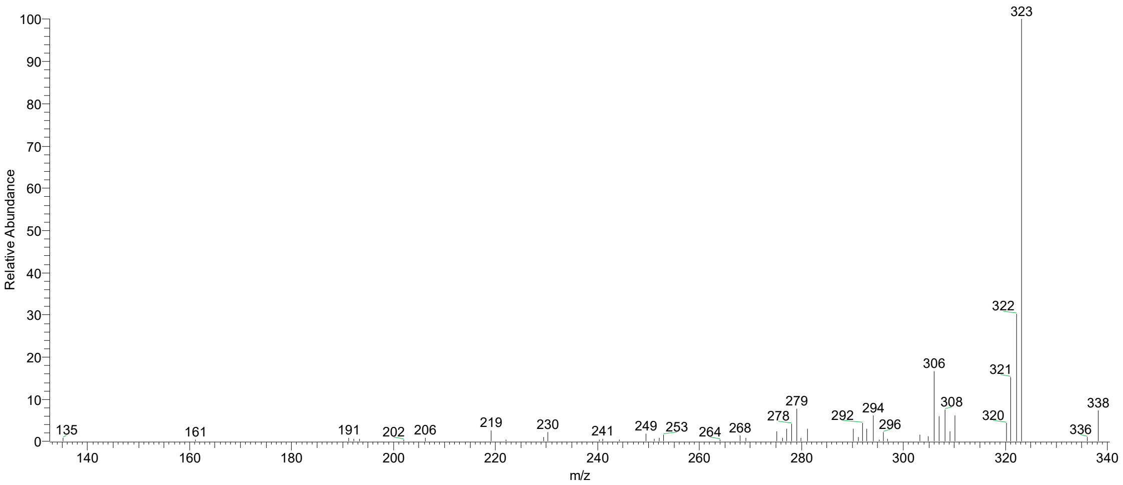

Figure S17. APCI-MS^n spectrum (positive mode) of ion *m/z* 338 (Subsessiline).

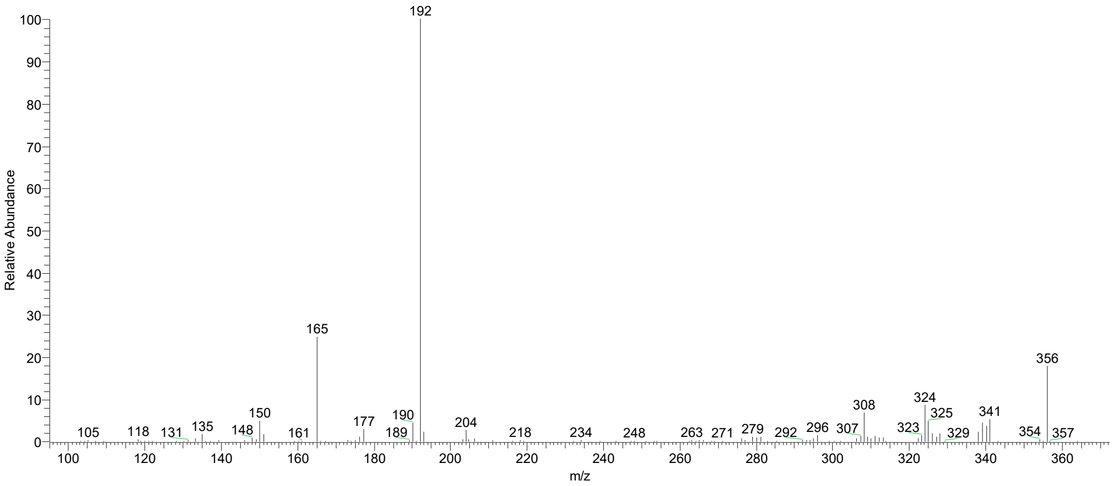

Figure S18. APCI-MS^n spectrum (positive mode) of ion *m/z* 356 (xylopinine).

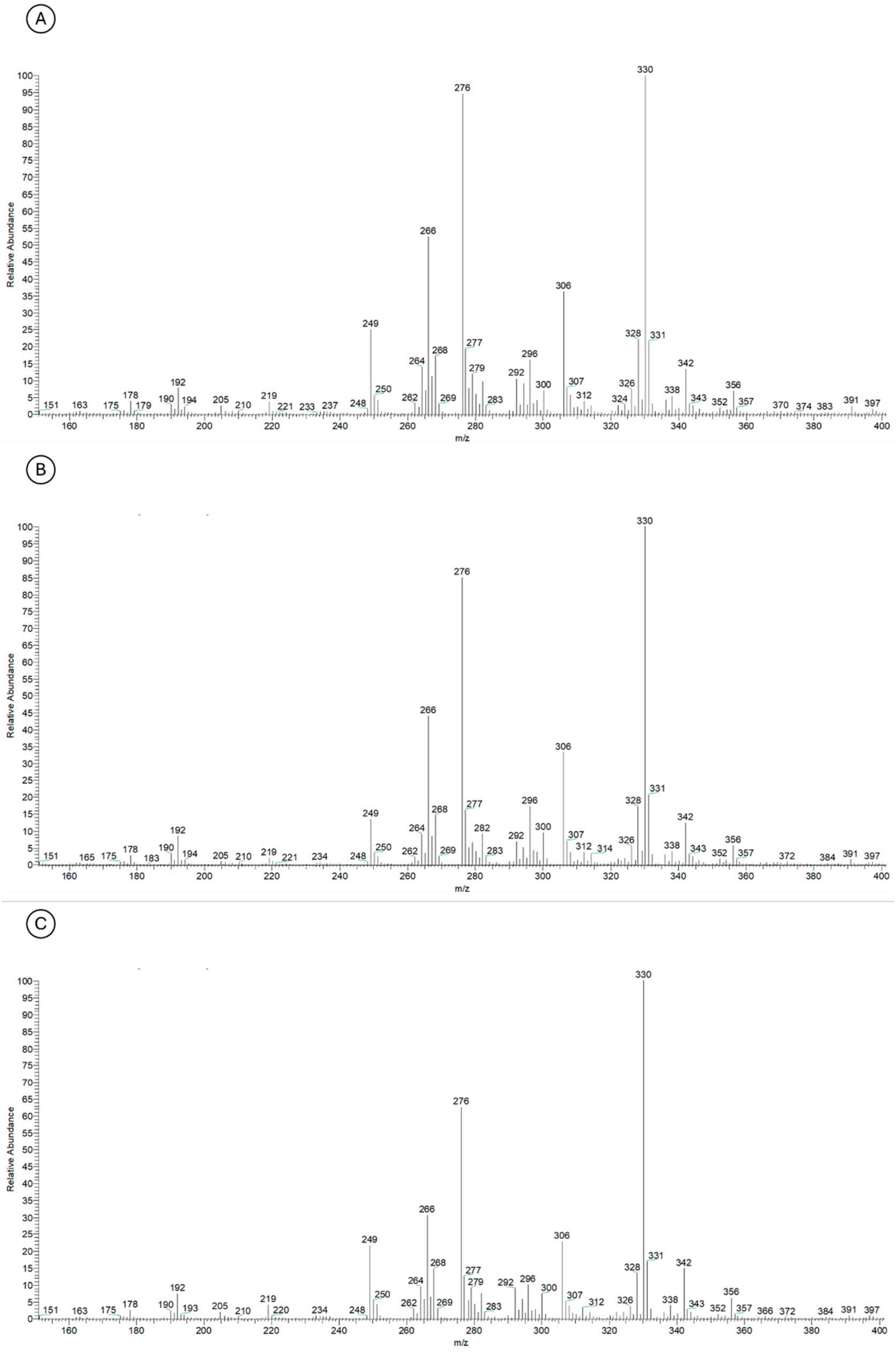

Figure S19. APCI-MS^n spectrum (positive mode) of total alkaloid extract from the root of ungrafted *Annona emarginata* without auxin supply (control) at 8 (A), 14 (B) and 20 (C) days after the beginning of the treatments (DAT).

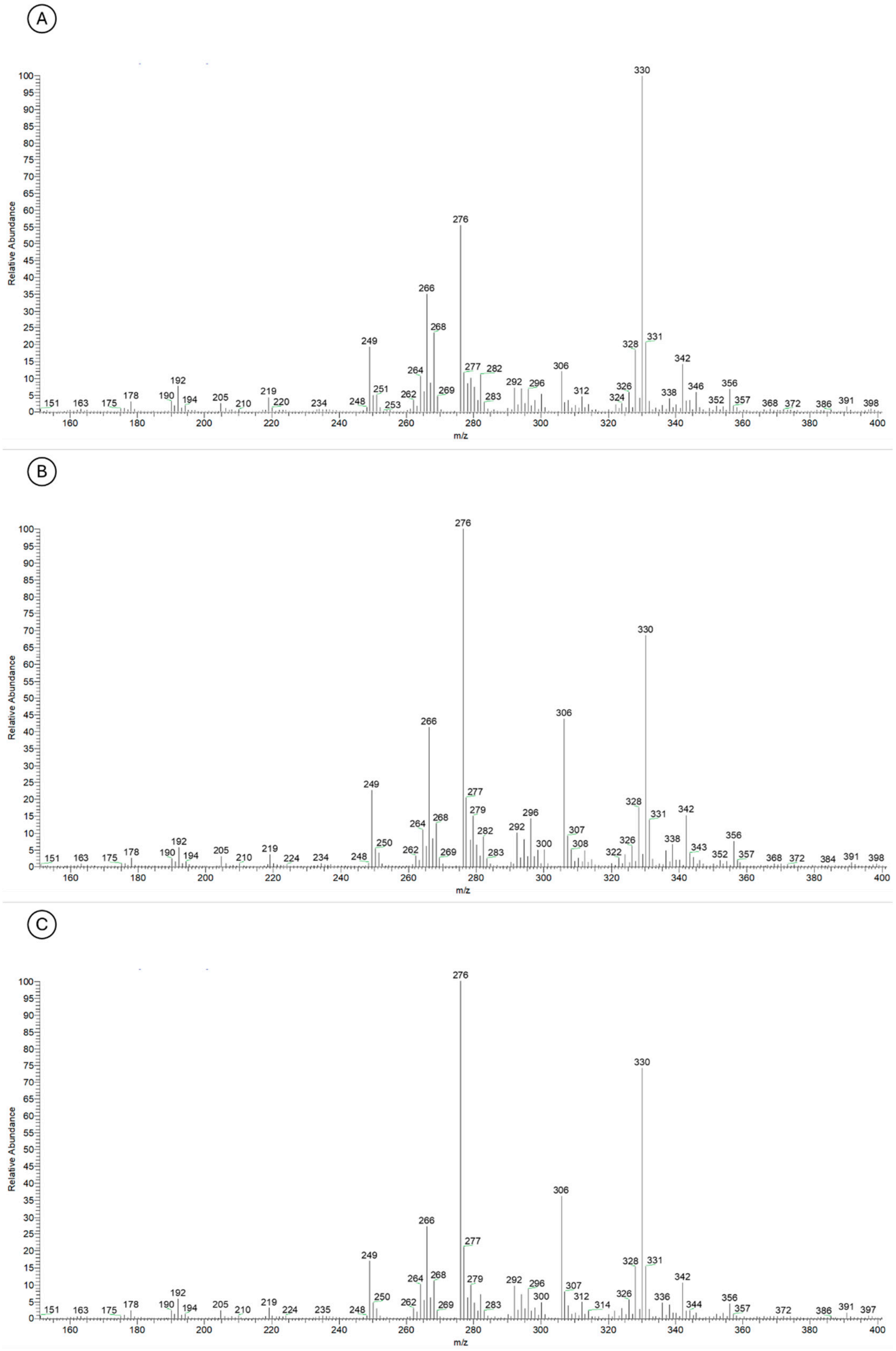

Figure S20. APCI-MS^n spectrum (positive mode) of total alkaloid extract from the root of ungrafted *Annona emarginata* treated with IAA at 8 (A), 14 (B) and 20 (C) DAT.

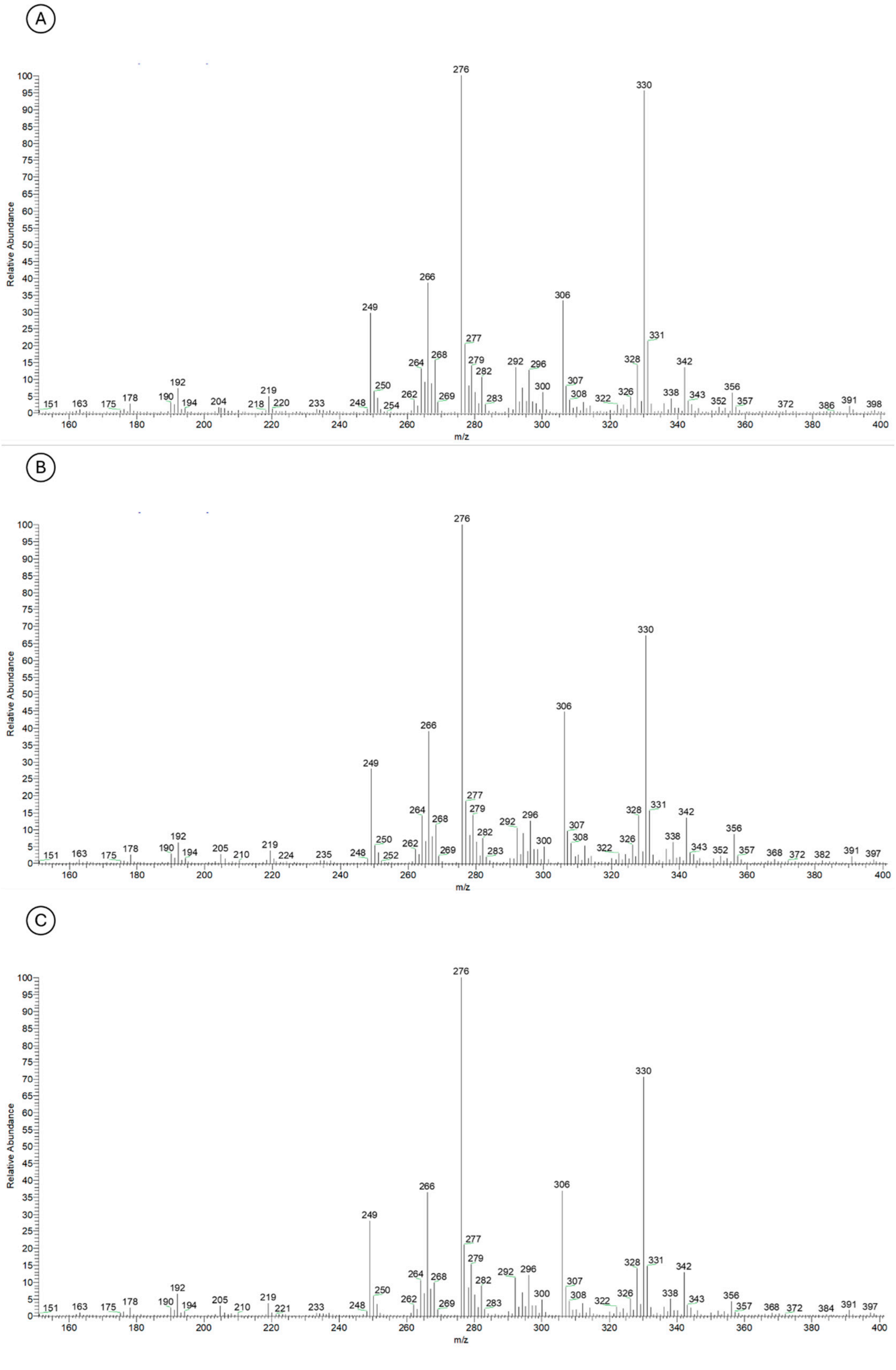

Figure S21. APCI-MS^n spectrum (positive mode) of total alkaloid extract from the root of ungrafted *Annona emarginata* treated with IBA at 8 (A), 14 (B) and 20 (C) DAT.

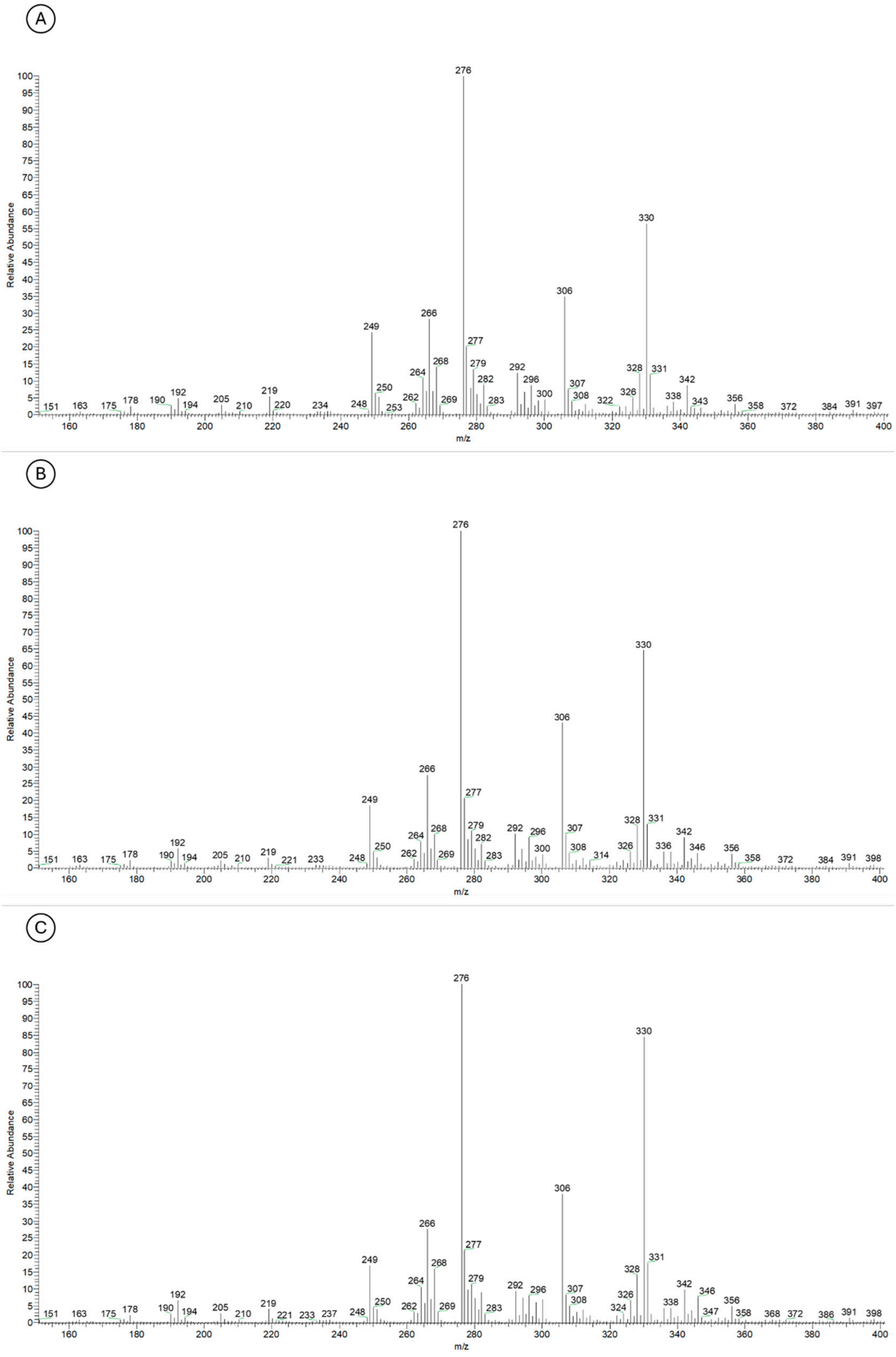

Figure S22. APCI-MS^n spectrum (positive mode) of total alkaloid extract from the root of ungrafted *Annona emarginata* treated with NAA at 8 (A), 14 (B) and 20 (C) DAT.

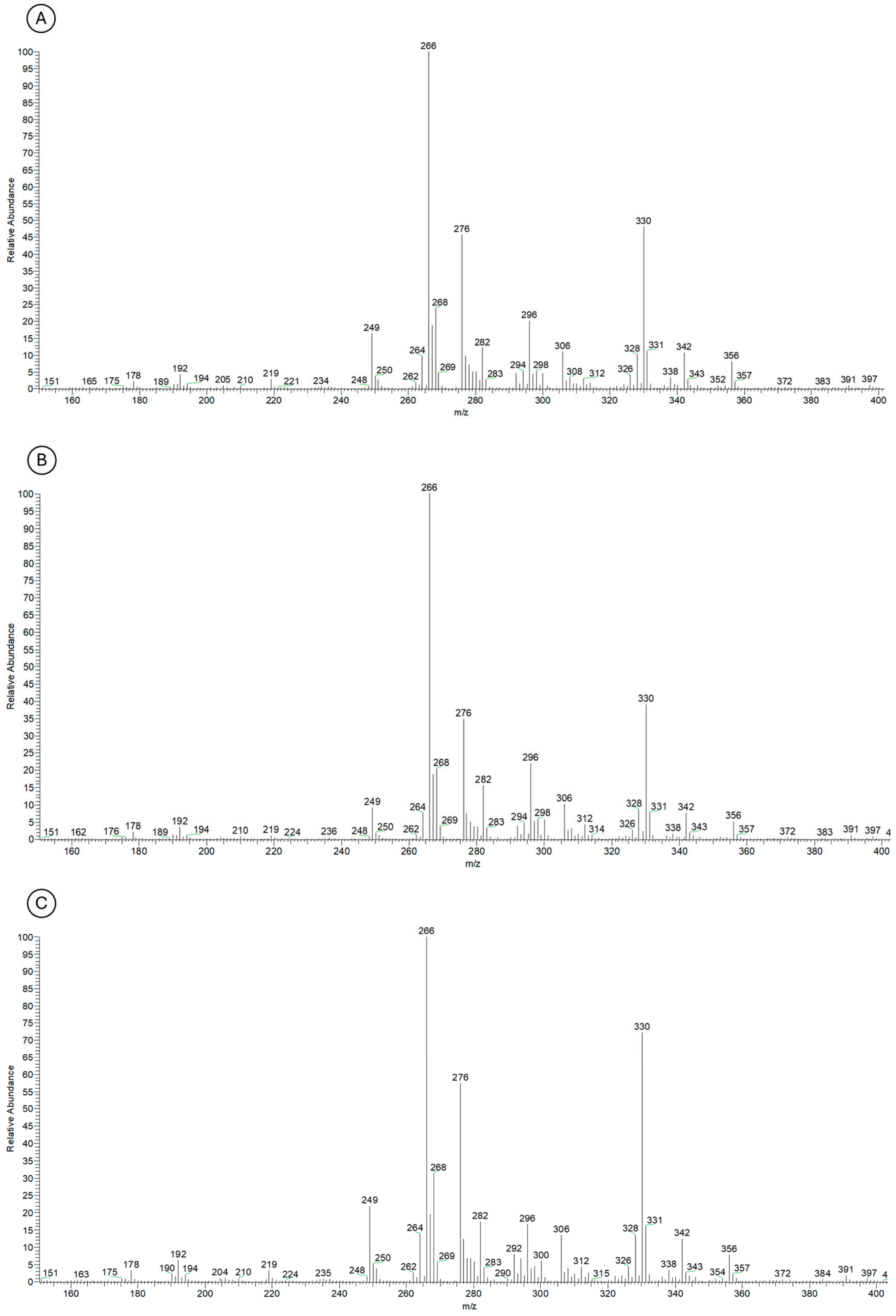

Figure S23. APCI-MS^{n} spectrum (positive mode) of total alkaloid extract from the root of *Annona emarginata* grafted with *Annona atemoya* without auxin supply (control) at 8 (A), 14 (B) and 20 (C) DAT.

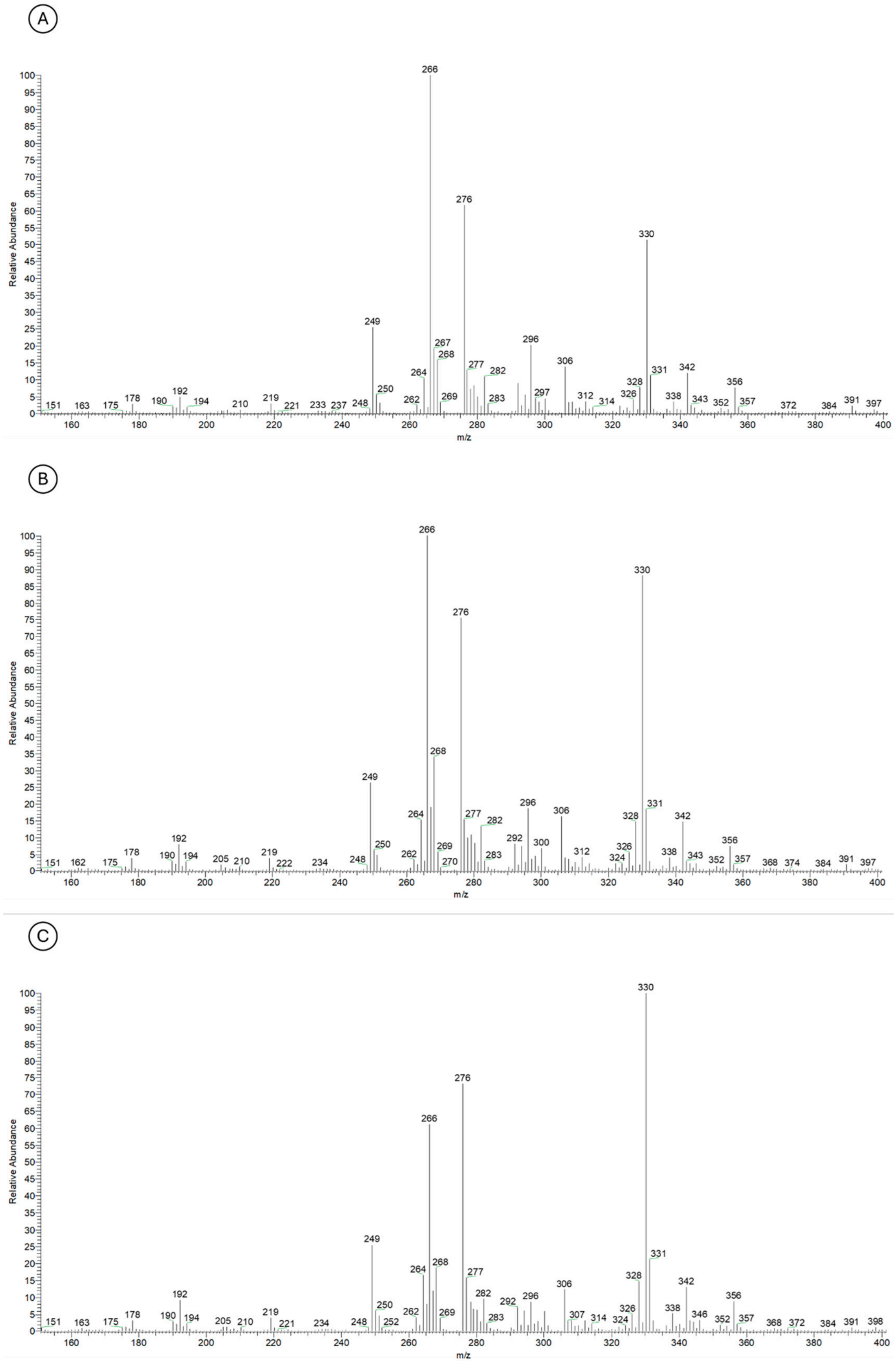

Figure S24. APCI-MS^n spectrum (positive mode) of total alkaloid extract from the root of *Annona emarginata* grafted with *Annona atemoya* treated with IAA at 8 (A), 14 (B) and 20 (C) DAT.

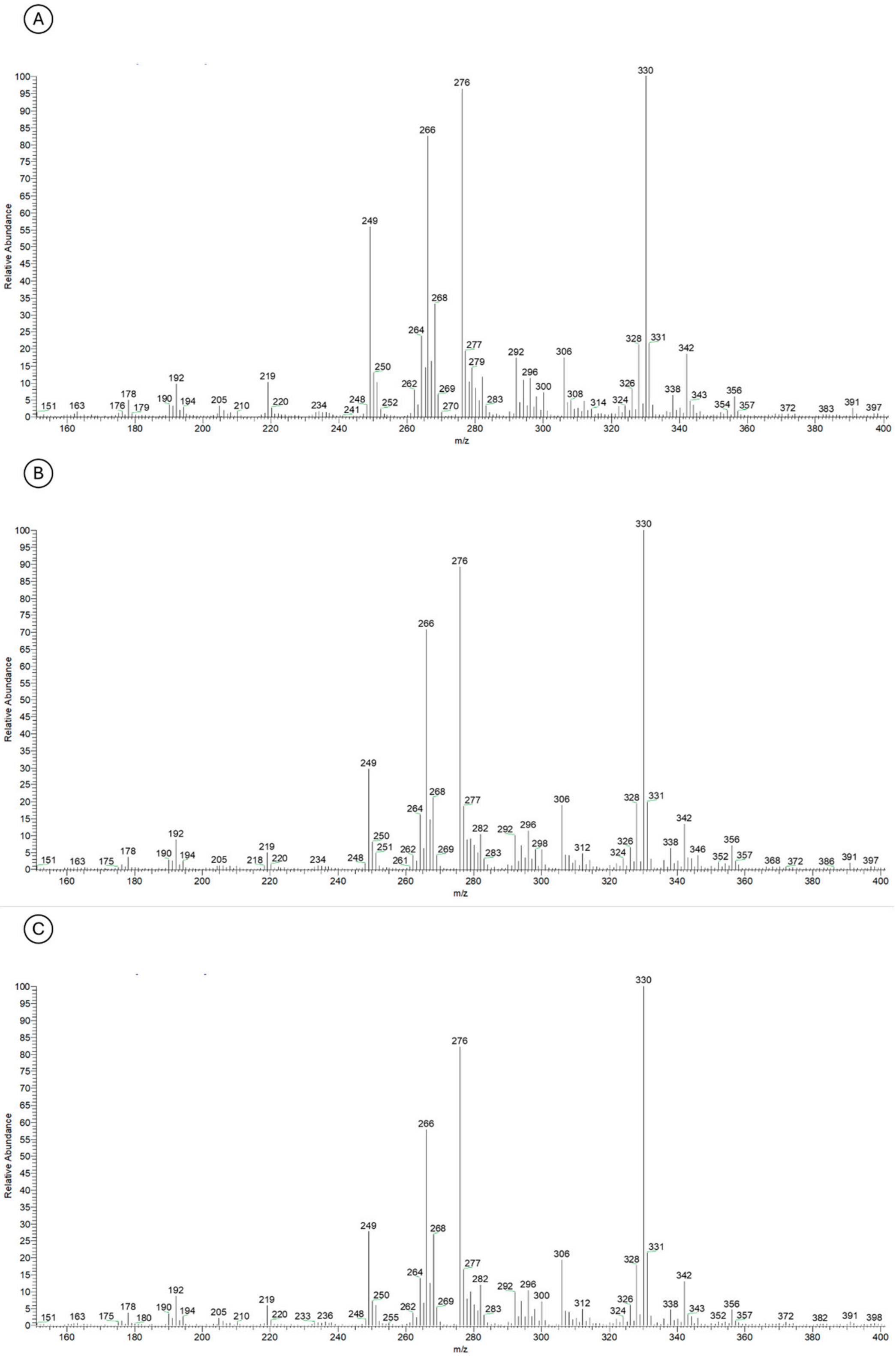

Figure S25. APCI-MS^n spectrum (positive mode) of total alkaloid extract from the root of *Annona emarginata* grafted with *Annona atemoya* treated with IBA at 8 (A), 14 (B) and 20 (C) DAT.

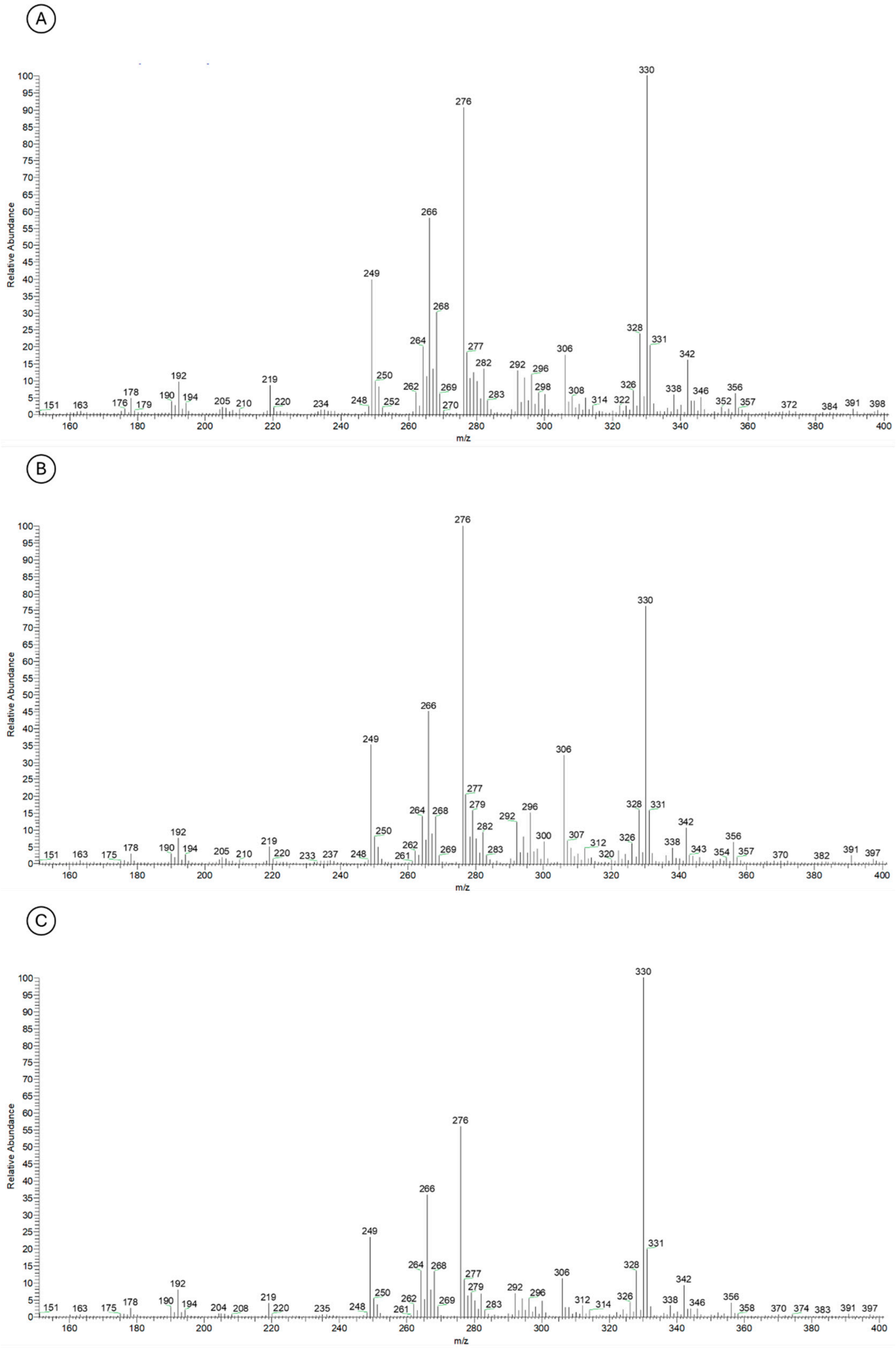

Figure S26. APCI-MS^n spectrum (positive mode) of total alkaloid extract from the root of *Annona emarginata* grafted with *Annona atemoya* treated with NAA at 8 (A), 14 (B) and 20 (C) DAT.

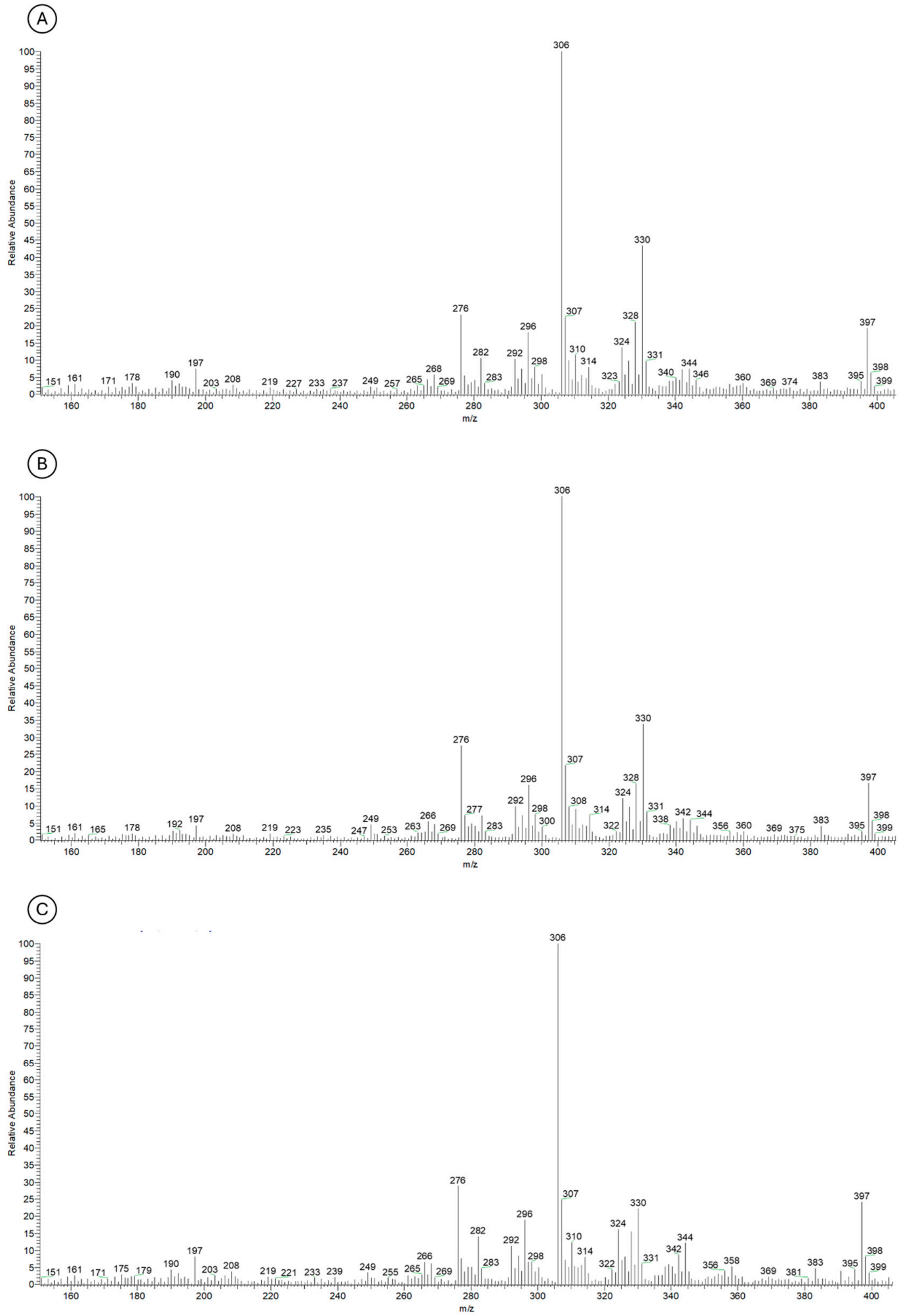

Figure S27. APCI-MS^n spectrum (positive mode) of total alkaloid extract from the leaves of ungrafted *Annona emarginata* without auxin supply (control) at 8 (A), 14 (B) and 20 (C) DAT.

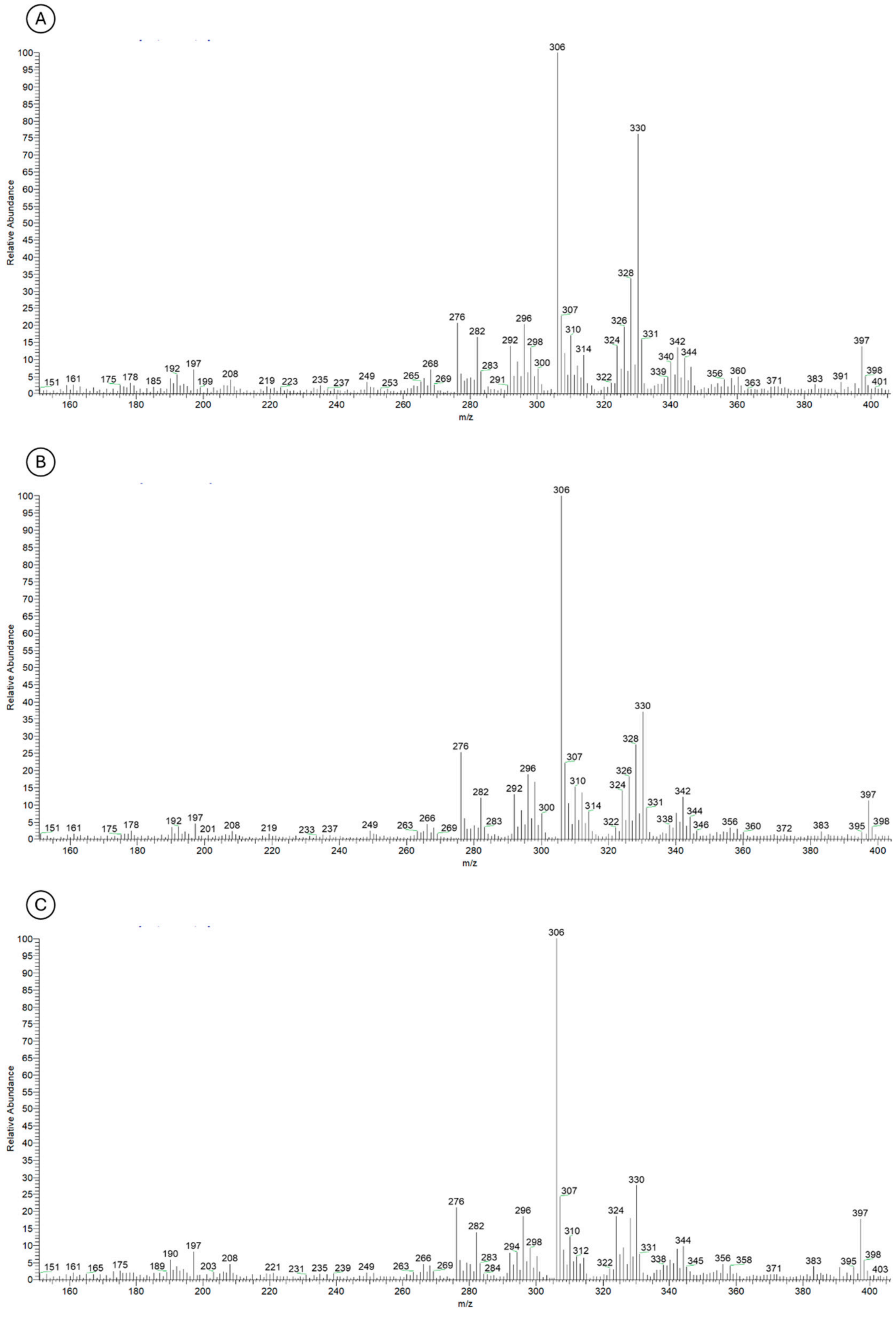

Figure S28. APCI-MS^n spectrum (positive mode) of total alkaloid extract from the leaves of ungrafted *Annona emarginata* treated with IAA at 8 (A), 14 (B) and 20 (C) DAT.

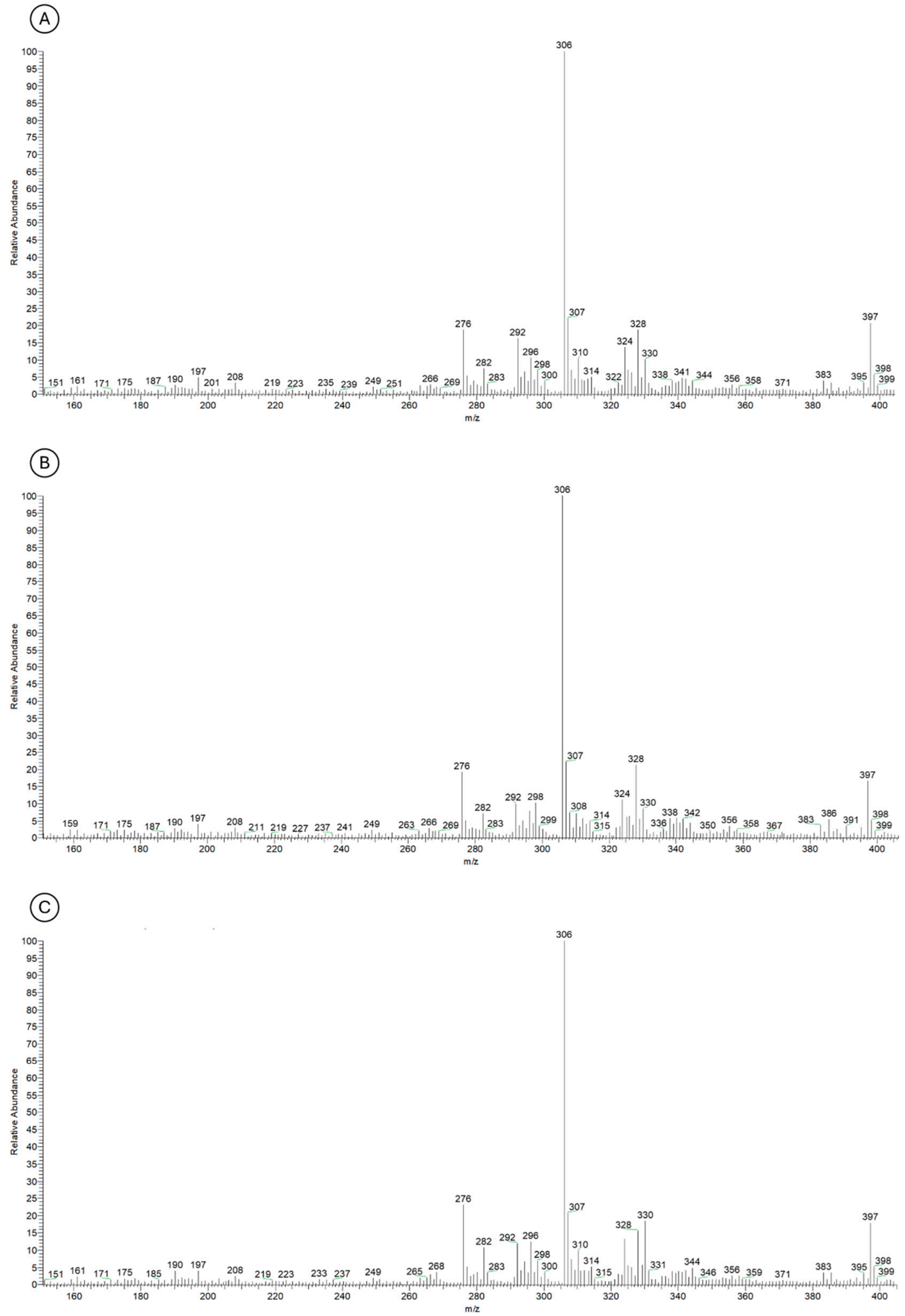

Figure S29. APCI-MS^n spectrum (positive mode) of total alkaloid extract from the leaves of ungrafted *Annona emarginata* treated with IBA at 8 (A), 14 (B) and 20 (C) DAT.

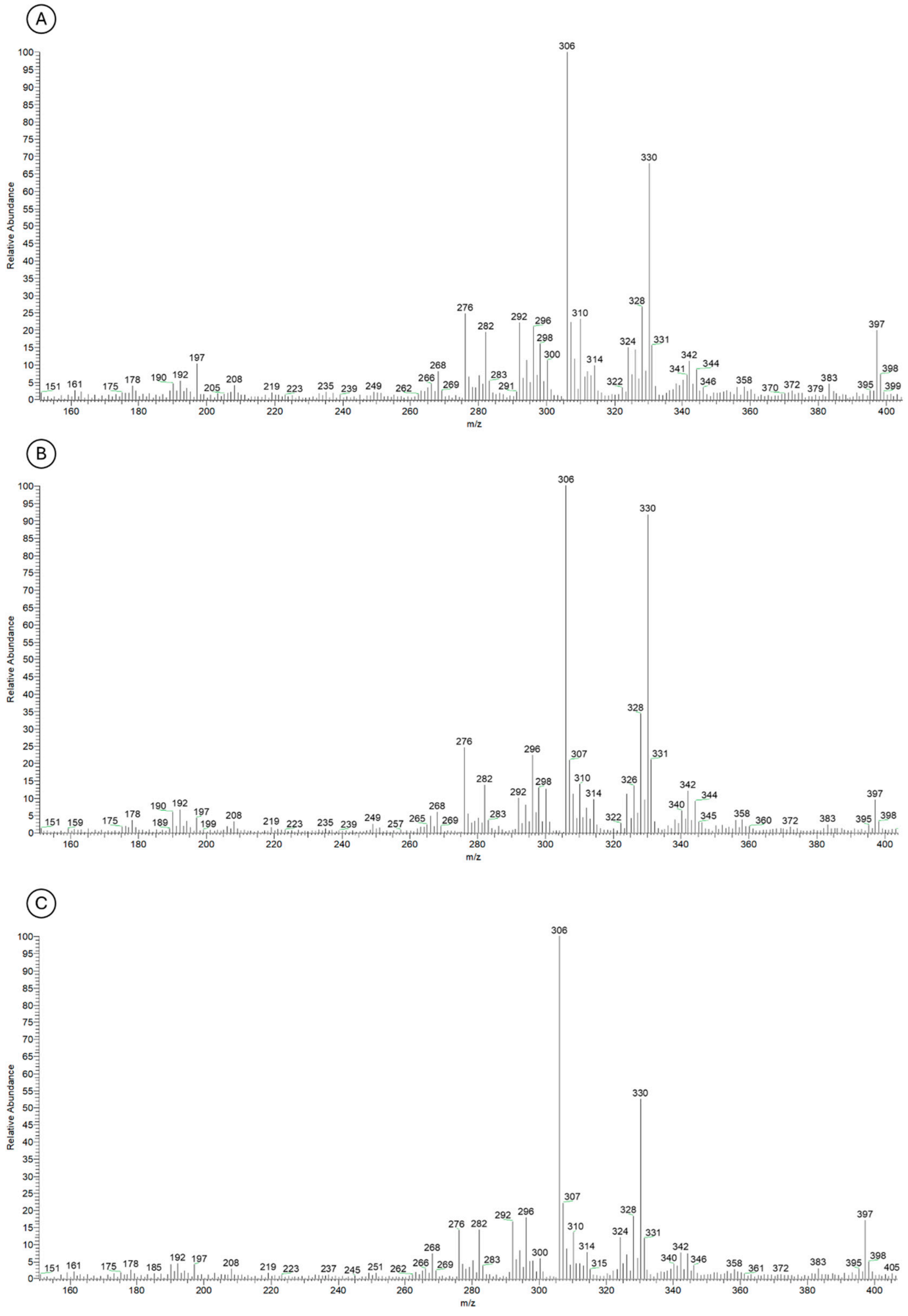

Figure S30. APCI-MS^n spectrum (positive mode) of total alkaloid extract from the leaves of ungrafted *Annona emarginata* treated with NAA at 8 (A), 14 (B) and 20 (C) DAT.

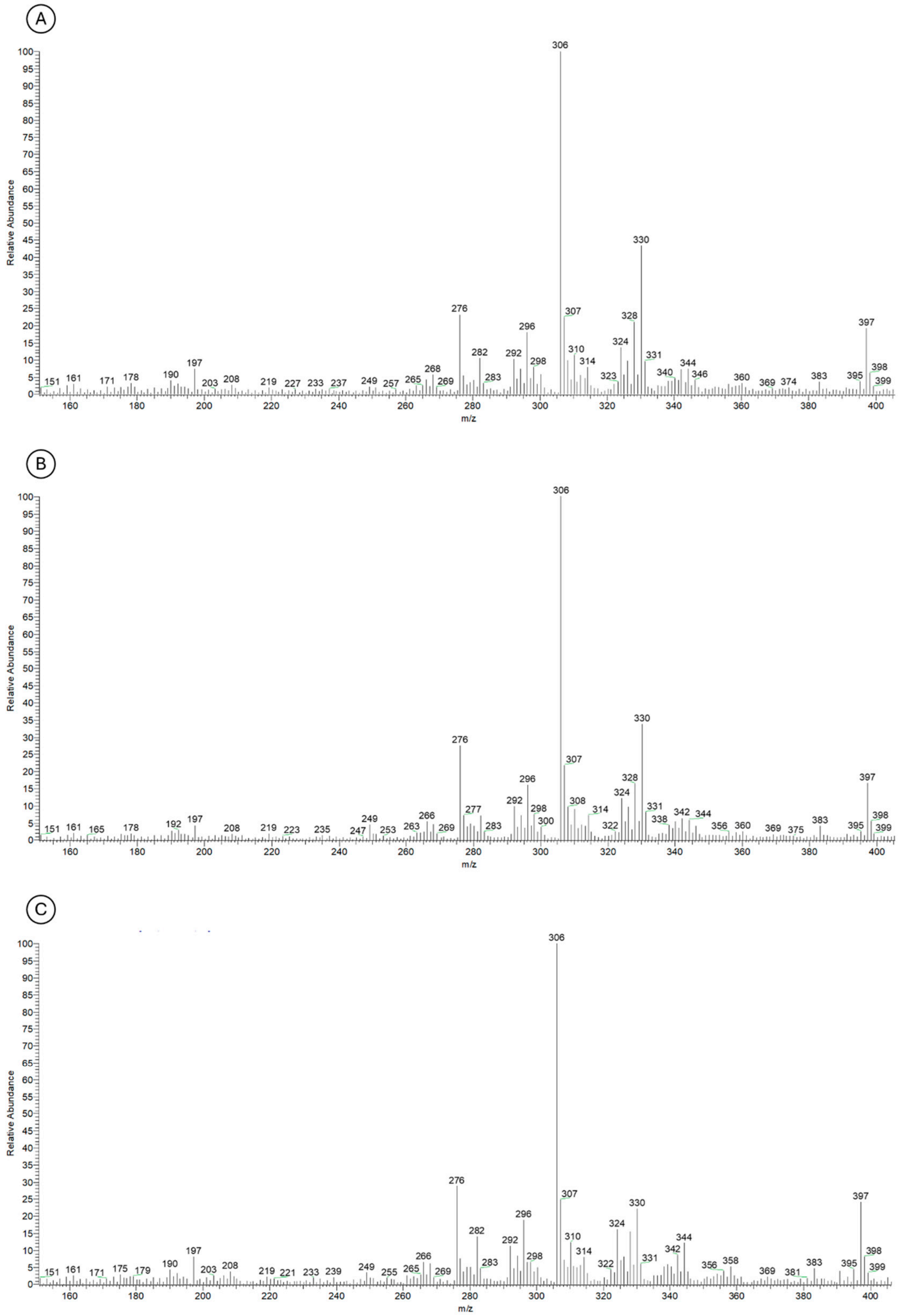

Figure S31. APCI-MS^n spectrum (positive mode) of total alkaloid extract from the leaves of *Annona atemoya* (grafted plants) without auxin supply (control) at 8 (A), 14 (B) and 20 (C) DAT.

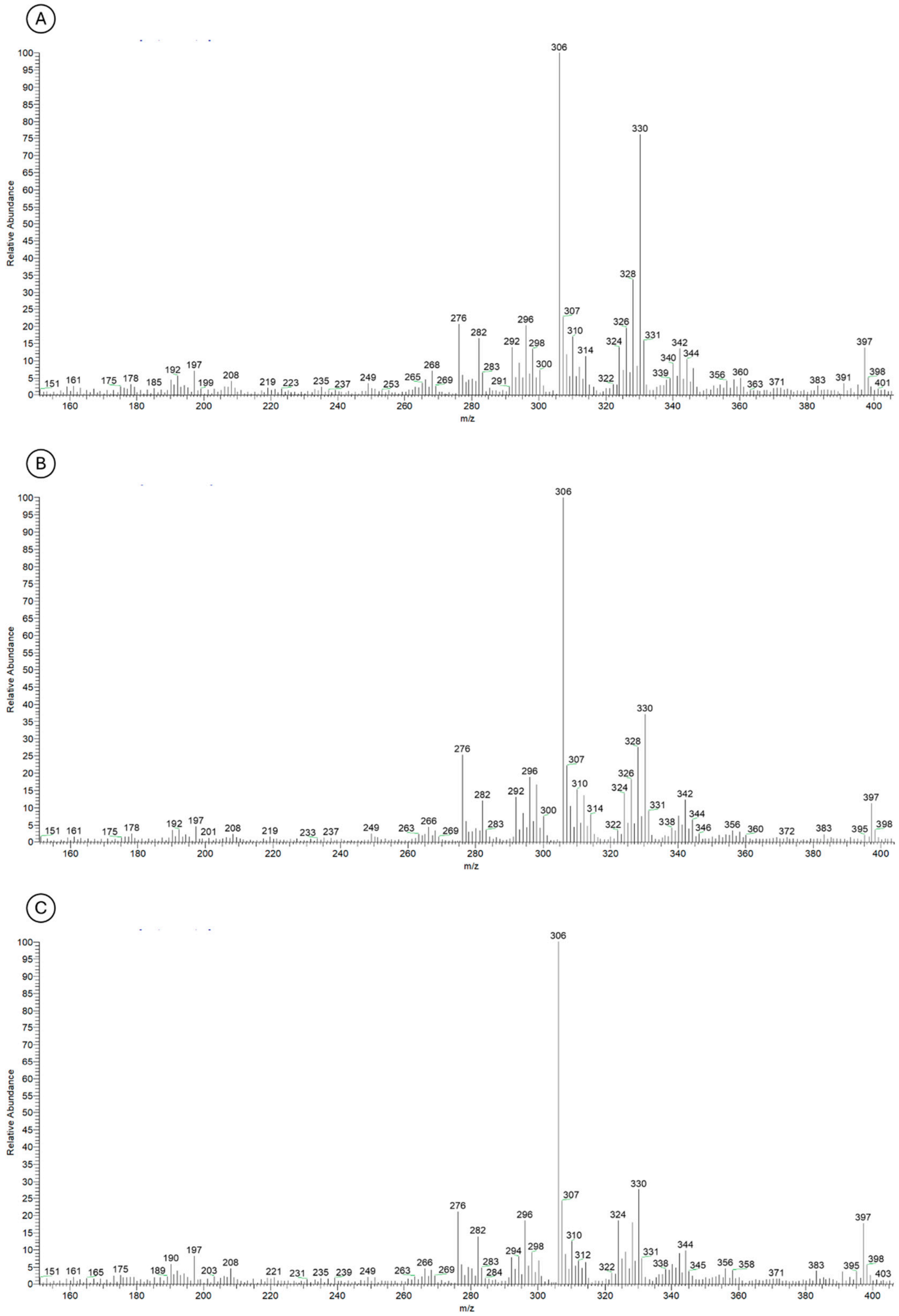

Figure S32. APCI-MS^n spectrum (positive mode) of total alkaloid extract from the leaves of *Annona atemoya* (grafted plants) treated with IAA at 8 (A), 14 (B) and 20 (C) DAT.

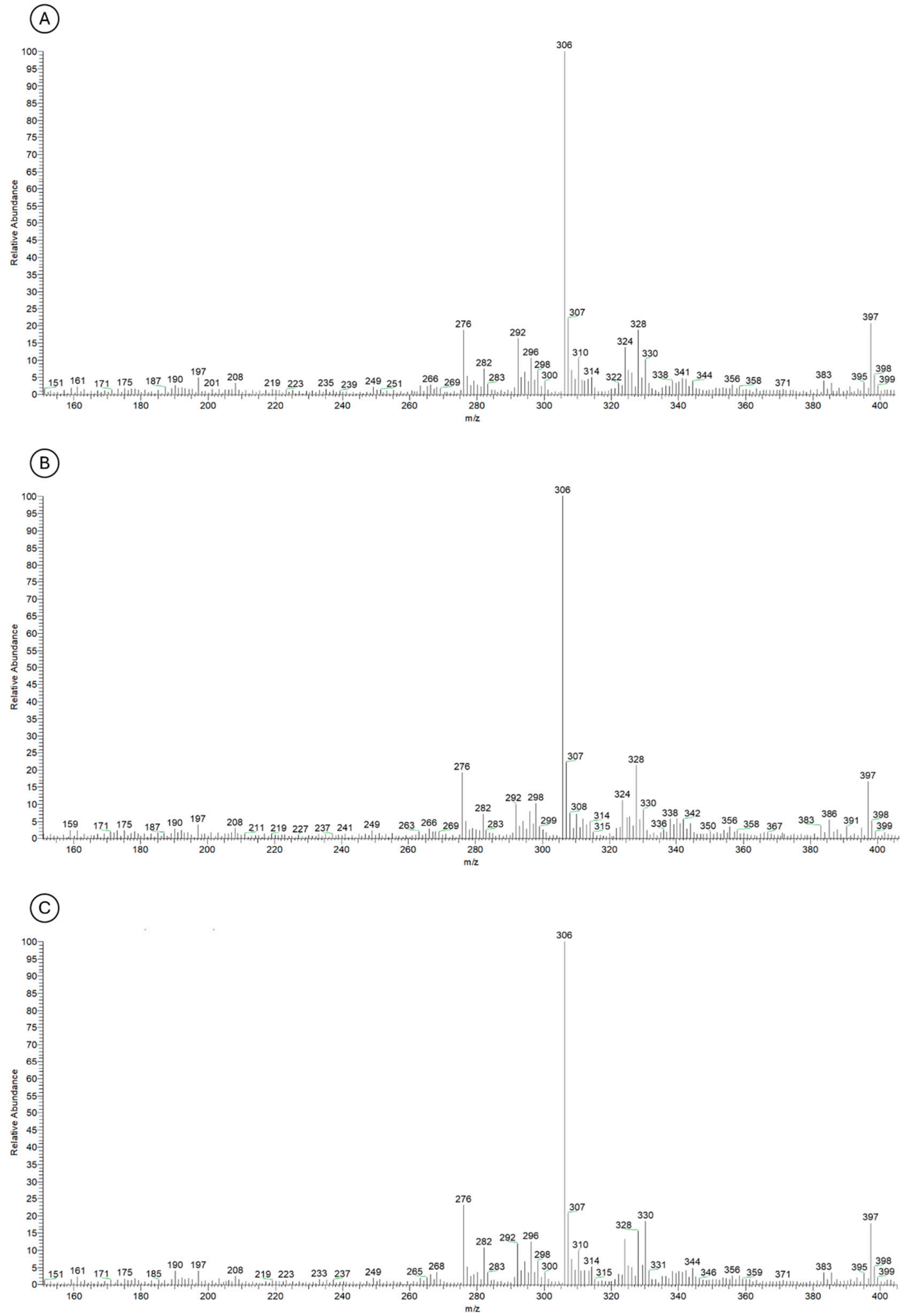

Figure S33. APCI-MS^n spectrum (positive mode) of total alkaloid extract from the leaves of *Annona atemoya* (grafted plants) treated with IBA at 8 (A), 14 (B) and 20 (C) DAT.

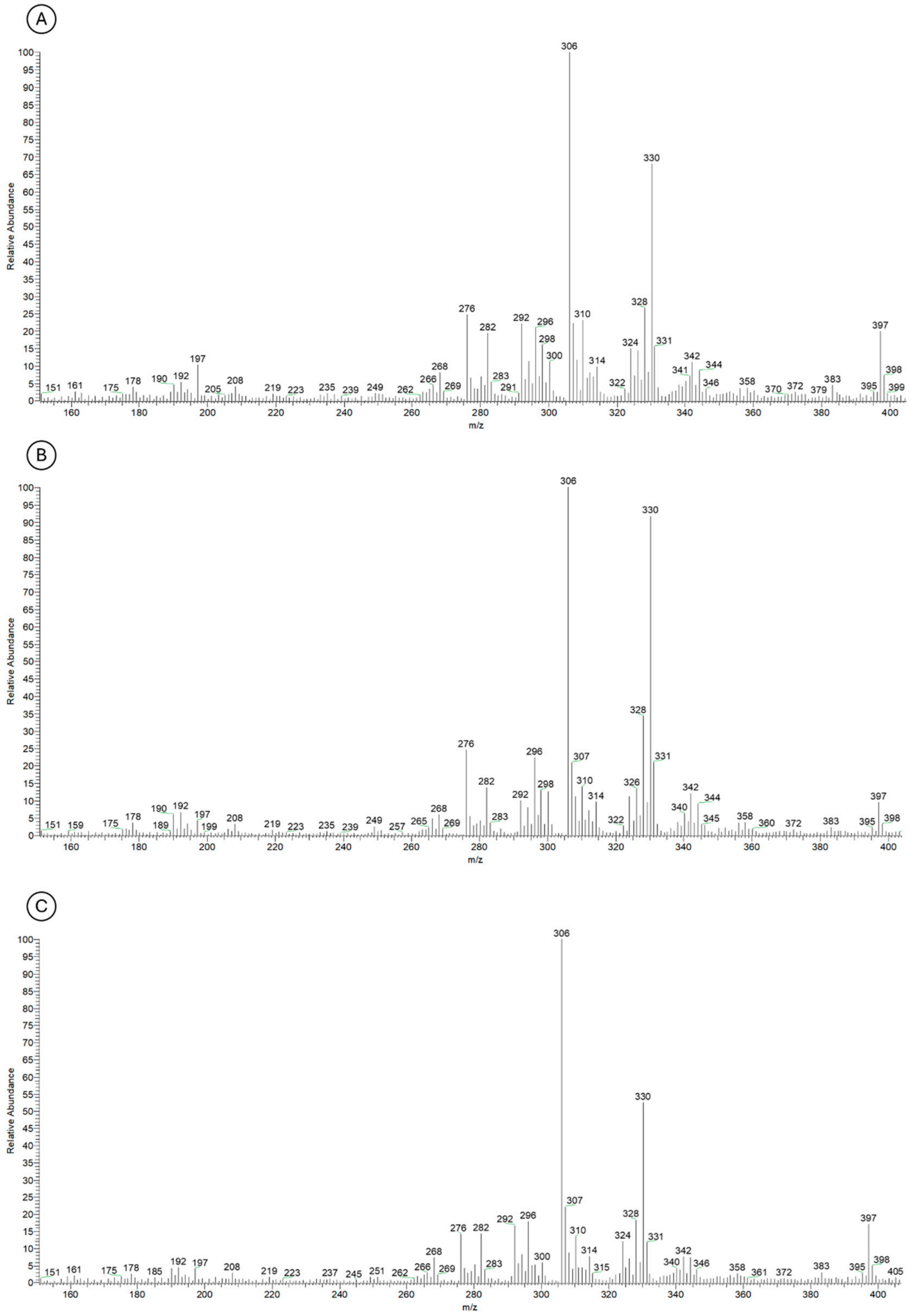

Figure S34. APCI-MS^n spectrum (positive mode) of total alkaloid extract from the leaves of *Annona atemoya* (grafted plants) treated with NAA at 8 (A), 14 (B) and 20 (C) DAT.

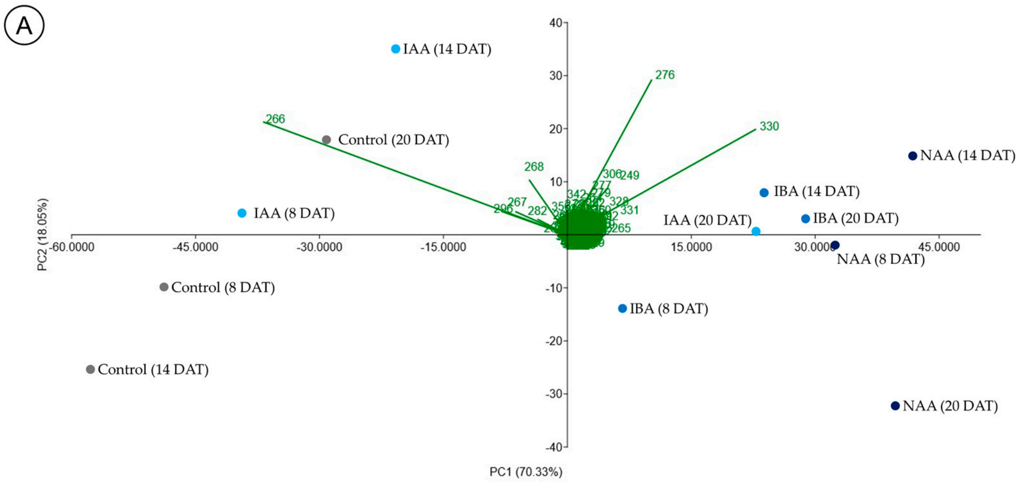

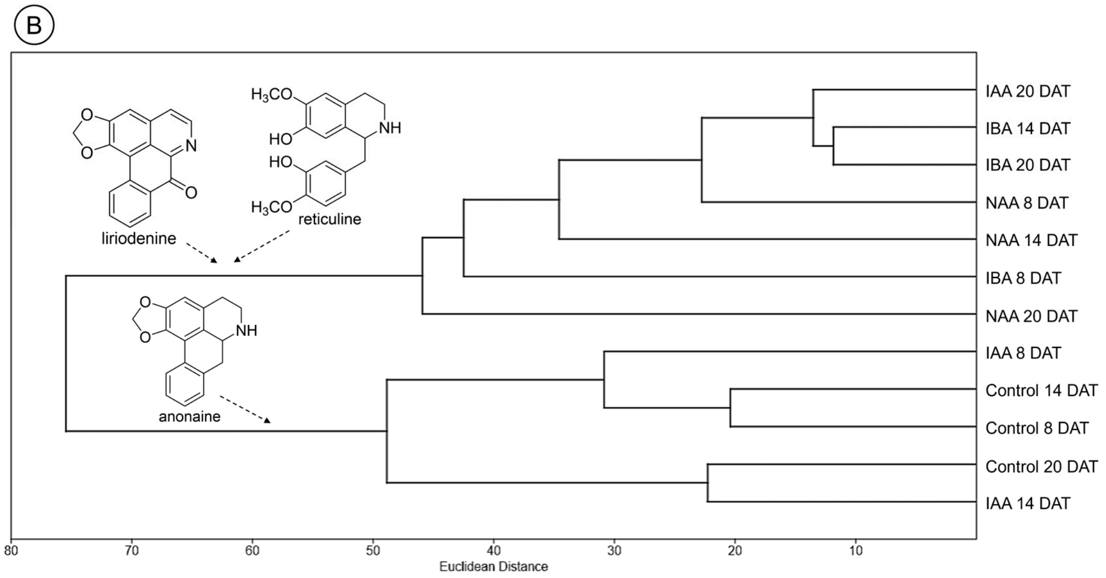

Figure S35. A) Principal Component Analysis (PCA) and B) Hierarchical Cluster Analysis (HCA) of alkaloids identified in *Annona emarginata* roots grafted with *Annona atemoya* treated with IAA, IBA and NAA in three collection times (8, 14 and 20 DAT) analyzed by APCI-MS.

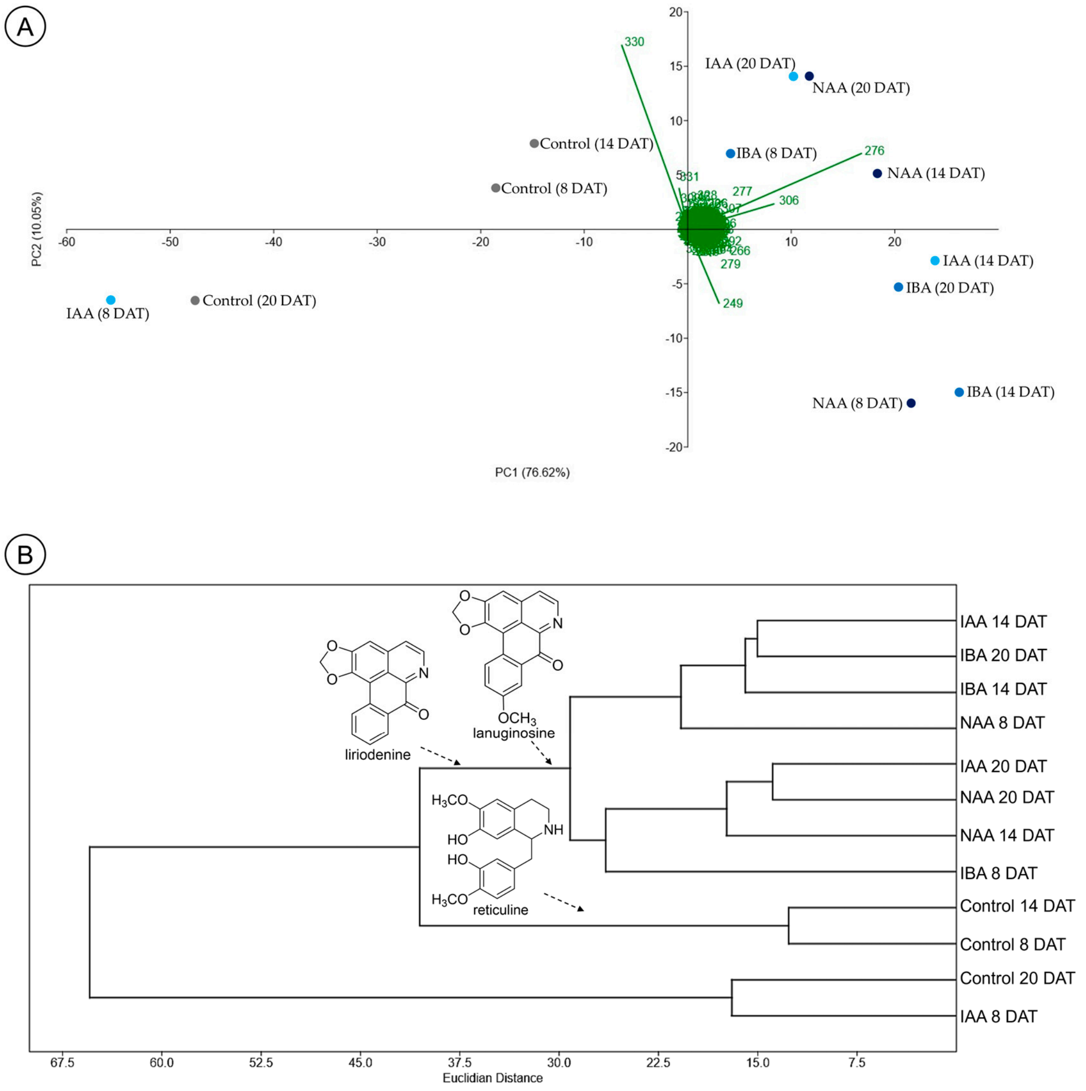

Figure S36. A) Principal Component Analysis (PCA) and B) Hierarchical Cluster Analysis (HCA) of alkaloids identified in the roots of ungrafted *Annona emarginata* submitted to treatments with IAA, IBA and NAA at three collection times (8, 14 and 20 DAT) analyzed by APCI-MS.
